# Enhancing the Electrocatalytic Oxidation of 5-Hydroxymethylfurfural Through Cascade Structure Tuning for Highly Stable Biomass Upgrading

**DOI:** 10.1007/s40820-024-01493-3

**Published:** 2024-08-22

**Authors:** Xiaoli Jiang, Xianhui Ma, Yuanteng Yang, Yang Liu, Yanxia Liu, Lin Zhao, Penglei Wang, Yagang Zhang, Yue Lin, Yen Wei

**Affiliations:** 1 School of Materials and Energy, University of Electronic Science and Technology of China, Chengdu, 611731 People’s Republic of China; 2 Hefei National Research Center for Physical Sciences at the Microscale, University of Science and Technology of China, Hefei, 230026 People’s Republic of China; 3 The Key Laboratory of Bioorganic Phosphorus Chemistry and Chemical Biology (Ministry of Education), Department of Chemistry, Tsinghua University, Beijing, 100084 People’s Republic of China; 4 School of Materials Science and Engineering, North Minzu University, Yinchuan, 750021 People’s Republic of China

**Keywords:** 5-Hydroxymethylfurfural oxidation reaction, Competitive adsorption, Cascade strategy, Elevated current density

## Abstract

Electrocatalytic 5-hydroxymethylfurfural oxidation reaction (HMFOR) provides a promising strategy to convert biomass derivative to high-value-added chemicals. Herein, a cascade strategy is proposed to construct Pd–NiCo_2_O_4_ electrocatalyst by Pd loading on Ni-doped Co_3_O_4_ and for highly active and stable synergistic HMF oxidation. An elevated current density of 800 mA cm^–2^ can be achieved at 1.5 V, and both Faradaic efficiency and yield of 2,5-furandicarboxylic acid remained close to 100% over 10 consecutive electrolysis. Experimental and theoretical results unveil that the introduction of Pd atoms can modulate the local electronic structure of Ni/Co, which not only balances the competitive adsorption of HMF and OH^–^ species, but also promote the active Ni^3+^ species formation, inducing high indirect oxidation activity. We have also discovered that Ni incorporation facilitates the Co^2+^ pre-oxidation and electrophilic OH* generation to contribute direct oxidation process. This work provides a new approach to design advanced electrocatalyst for biomass upgrading.

**Supplementary Information:**

The online version contains supplementary material available at 10.1007/s40820-024-01493-3.

## Highlights

- A novel cascade strategy is proposed to construct Pd-NiCo_2_O_4_ electrocatalyst.
- First time discovery that Ni incorporation together with Pd loading is able to balance the competitive adsorption of OH^–^ and 5-hydroxymethylfurfural.
- Pd–NiCo_2_O_4 _promotes both the indirect and direct synergistic oxidation process.
- Pd–NiCo_2_O_4_ catalyst exhibits extraordinary current density and excellent Faradaic Efficiency at a low potential.

**Supplementary Information:**

The online version contains supplementary material available at 10.1007/s40820-024-01493-3.

## Graphical abstract

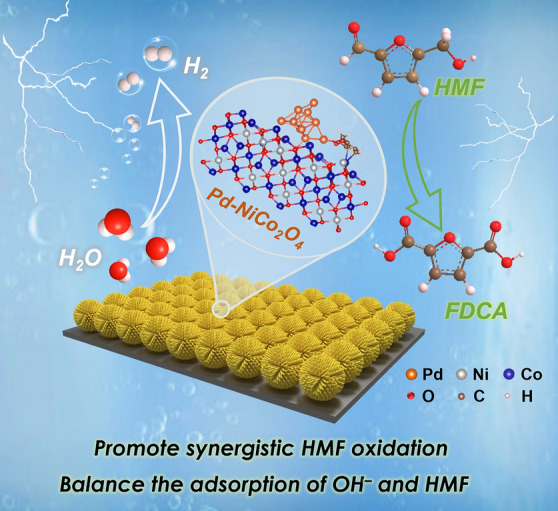

## Introduction

The massive consumption of fossil energy has led to excessive carbon dioxide emissions and plastic waste pollution, shifting the over-reliance on fossil energy to a carbon neutral economy rooted in green and sustainable development [1–3]. Electrocatalytic water splitting is a promising and efficient technology to alleviate the increasingly serious energy crisis, but the slow kinetic anodic oxygen evolution reaction (OER) seriously restricts the wide application [4–6]. In this context, hybrid water electrolysis is a more technically and economically feasible alternative to OER. Electrocatalytic conversion of renewable biomass resources provides a feasible and attractive approach to generate high value-added chemicals and lower cell voltage [7, 8]. 5-Hydroxymethylfurfural (HMF) is an important and impressive biomass derivative that can be selectively oxidized or reduced to produce polymer monomers, fine chemicals, and liquid fuels [9]. One such oxidation product, 2,5-furanodicarboxylic acid (FDCA) is particularly important. Because of its similar structure and properties to the petroleum-based monomer terephthalic acid (PTA), FDCA is considered a key precursor for the preparation of green and biodegradable bio-based polyesters as a substitute for PTA [10, 11]. By coupling electro-oxidation HMF with hydrogen evolution reaction (HER), high value-added FDCA and hydrogen energy can be acquired, which offers an environmentally sustainable alternative with high energy efficiency [12].

Electrocatalytic 5-hydroxymethylfurfural oxidation reaction (HMFOR) is an environmentally friendly method at ambient temperature and pressure [13]. Regardless of the conventional indirect oxidation or potential-dependent (PD) oxidation mechanism, catalytic conversion of HMF inevitably requires adsorption of the reactive species [14, 15]. In the process of HMFOR under alkaline conditions, it requires the simultaneous participation of organic molecules and OH^−^ species, where the competitive adsorption between OH^−^ and HMF molecules occurs on catalysts affecting the HMFOR activity significantly [16, 17]. To achieve high conversion efficiency and catalytic activity, the catalyst needs to possess appropriate adsorption capabilities for both HMF and OH^−^. However, due to limited adsorption sites, these two substrates will compete for adsorption on the catalyst surface, highlighting the importance of balancing the adsorption of HMF and OH^−^ for enhancing catalytic performance. Therefore, great efforts should be devoted to exploring efficient electrocatalysts to balance the adsorption competition of HMF and OH^−^ on the catalyst surface.

Spinel oxides (AB_2_O_4_) is a classical transition metal oxides (TMO) with A^2+^ and B^3+^ cations occupying centers of tetrahedron (Td) and octahedron (Oh), respectively [18, 19]. Owing to its abundant active sites and tunable coordination structures, efficient strategies have been applied for optimizing intrinsic catalytic performance [7, 20]. Zou et al. revealed that the tetrahedral Co site contributes to the adsorption of HMF, while the octahedral Co site tends to adsorb the OH^−^ filled into the oxygen vacancy to improve the reaction kinetics [21]. Zang’s group found that Mo doping modulated the electronic structure of Co_3_O_4_, enhancing the metal–oxygen bonding strength and promoting the CoOOH active substances formation [22]. Zou et al. also found that the Co^3+^_Oh_ in Co_3_O_4_ is catalytic site, and the Co^2+^_Td_ is adsorption site and the introduction of copper ions at the tetrahedral site enhanced the adsorption of HMF on the catalyst surface [23]. HMFOR activity can be further improved by partial substituting tetrahedral sites in Co_3_O_4_ with Ni. Nickel substitution could modify the crystal structure and coordination environment of cobalt, which can enhance the reactivity of hydroxyl and aldehyde groups [24]. Nevertheless, the HMFOR activity of spinel-based electrocatalysts mentioned above, including the onset potential and large current density still needs to be improved. More importantly, transition metal oxides often exhibit strong OH^−^ adsorption ability in strong alkaline solutions, leading to weaker adsorption of HMF [25]. Therefore, regulating the selective adsorption of metal oxides to HMF and intermediates is crucial for HMFOR.

Herein, we present, for the first time, an integrated system of Pd-NiCo_2_O_4_ electrocatalyst on Ni foam (NF) by Ni substitution of Co in Co_3_O_4_ to form NiCo_2_O_4_ followed by Pd-loading uniformly on NiCo_2_O_4_ for select oxidation of HMF to FDCA. The partial substitution of the octahedron Co^3+^ in Co_3_O_4_ by Ni promotes the structural evolution, inducing the formation of oxygen vacancies and redox active Co^3+^ species, facilitating the direct oxidation process. Density functional theory (DFT) calculations validate the key role of Pd introduction for balancing the competitive adsorption of HMF and OH^−^ on the electrode surface, which achieves Ni/Co electronic structure tuning. In addition, Pd loading reduces the pivotal reaction barrier step of 5-hydroxymethyl-2-furancarboxylic acid (HMFCA) dehydrogenation to 5-formyl-2-furancarboxylic acid (FFCA). In situ Raman measurements disclose that the formation of Ni^2+^/Ni^3+^ redox active species on Pd-NiCo_2_O_4_ electrode promotes the indirect oxidation process. Consequently, the optimized Pd-NiCo_2_O_4_ displays elevated current density (*E*_800_ = 1.5 V) and can effectively electrooxidize HMF to FDCA at the current density of 200 mA cm^−2^, with a high Faradaic efficiency of 99.6%. Notably, stable continuous electrolysis up to ten cycles can be achieved and maintained the FE of approximately 99%. The integrated electrocatalytic system merely requires voltage of 1.51 V to reach current density of 100 mA cm^–2^ and the Faradaic efficiency remained highly stable throughout continuous electrolysis. This work demonstrates the significance of balancing the competitive adsorption and the synergistic oxidation providing inspiration for the design and construction of effective electro-catalysts.

## Experimental Section

### Preparation of Materials

NiCo_2_O_4_ nanosheets were prepared according to the previous reports with slight modifications [26]. Firstly, Ni foam (NF, 3 cm × 4 cm) was first treated with a mixture solution of concentrated HCl and deionized water (volume ratio 1:3) under ultrasonication for 15 min to remove surface oxide layer, following by washing the NF with ultrapure water and ethanol several times until pH≈7, respectively. The cleaned NF was then immersed into a 40 mL aqueous solution containing 1 mmol Ni(NO_3_)_2_·6H_2_O, 2 mmol Co(NO_3_)_2_·6H_2_O, 4 mmol CO(NH_2_)_2_ and 2 mmol NH_4_F. The mixed system was then sealed and maintained at 120 °C for 8 h in a blast drying oven to obtain the NiCo-hydroxide. After annealing at 350 °C for 2 h under air atmosphere, NiCo_2_O_4_/NF was obtained. The coating amount of NiCo_2_O_4_ was about 2.46 mg cm^−2^.

The Pd loaded NiCo_2_O_4_ was prepared via an impregnation method. Typically, the as-prepared NiCo_2_O_4_/NF (1 cm × 3 cm) was immersed into a palladium chloride ethanol solution with different concentration (0.5, 1, and 1.5 mg mL^−1^) and maintained under 30 °C for 140 min. The obtained materials are denoted as Pd–NiCo_2_O_4_-x (x = 1, 2, 3, corresponding to 0.5–1.5 mg mL^−1^). The mass loadings of Pd–NiCo_2_O_4_-x were about 1.93 mg cm^−2^. Unless otherwise stated, all the involved Pd-NiCo_2_O_4_ in this work refers to Pd-NiCo_2_O_4_-2.

The preparation of Co_3_O_4_ was similar to NiCo_2_O_4_ except without the addition of Ni resource, with the mas loading about 2.48 mg cm^−2^. And the Pd/NF was prepared by immersing the pure Ni foam into a 1 mg mL^−1^ palladium chloride ethanol solution and maintaining the same reaction conditions as Pd–NiCo_2_O_4_. The mass loading of Pd/NF was about 1.68 mg cm^−2^.

### Materials Characterizations

The morphologies of the catalysts were characterized by field emission scanning electron microscopy (FE-SEM, Thermo Fisher TALOS F200X, America) and transmission electron microscopy (TEM) and atomic-resolution high-angle annular dark-field scanning transmission electron microscopy (HAADF-STEM) images equipped with an energy-dispersive spectrometer (EDS) were performed on JEM-ARM 200F with an accelerating voltage of 200 kV). The X-ray absorption fine structure data were collected at the Rapid XAFS 1 M Plus. The chemical composition and surface states were analyzed by X-ray diffraction (XRD, Bruker D8 ADVANCE) and X-ray photoelectron spectroscopy (XPS, Thermo Scientific K-Alpha), respectively. For the determination of vacancy, powder samples were taken and tested directly in a quartz sample tube of the electron paramagnetic resonance (EPR) instrument. EPR spectra were acquired on the Bruker EMXnano instrument with a central field of 3000 G, a scanning range of 4000 G, a microwave frequency of 9.60 GHz, and a conversion time of 25 ms.

### Electrochemical Measurements

Electrochemical experiments were performed using a CHI 760E electrochemical workstation within a conventional three-electrode setup at ambient temperature. The electrodes comprised the synthesized catalyst (1 cm × 0.5 cm) as the working electrode, a carbon rod as the counter electrode, and Hg/HgO as the reference electrode. Electrochemical evaluations involving HMF were executed under constant stirring. The reported potentials were adjusted to the reversible hydrogen electrode (RHE) scale using the formula: E_(RHE)_ = E_(Hg/HgO)_ + 0.059pH + 0.098. Linear sweep voltammetry (LSV) tests for the oxidation of HMF were performed at a 5 mV/s scan rate in a 1 M KOH solution containing 50 mM HMF, in a single-compartment cell. To account for the ohmic potential drop due to solution resistance, 90% iR-compensation was utilized. The electrocatalytic experiments of different samples, potentials, and cycle tests of Pd–NiCo_2_O_4_ for HMFOR were carried out in a divided H-type cell without iR-correction, separated by a Nafion 117 proton exchange membrane, evaluating by chronoamperometry test in 10 mL 1 M KOH with 50 mM HMF. All mentioned electrochemical procedures were conducted with ongoing agitation, unless specified otherwise. As for the two-electrode system, Nafion 117 membrane was used as the proton exchange membrane in a divided H-type cell and Pd–NiCo_2_O_4_ (effective area of 1 cm^2^) served as both the cathode and anode electrode for HMFOR and HER. The double-layer capacitance (*C*_dl_) was measured at different scan rates within the potential range of − 0.2 to − 0.1 V vs. Hg/HgO to determine the electrochemical surface area (ECSA). The ECSA of the samples was calculated using the following formula: ECSA = *C*_dl_/*C*_s_, where *C*_s_ = 0.040 mF cm^−2^ is the specific capacitance of the sample in alkaline electrolyte based on typical reported value [27].

### In Situ Raman Spectrum Tests

In situ Raman spectroscopy was conducted using a confocal Raman microscope (Alpha300R, WITEC, Germany) equipped with a 532 nm laser, in conjunction with the CHI 760E electrochemical workstation. The setup of in situ Raman consisted of a Teflon shell, a quartz glass plate, and a glassy carbon electrode. The 532 nm semiconductor laser was focused using a 50 × objective lens to vertically illuminate the sample. A three-electrode configuration was employed, with the catalyst-coated working electrode, Ag/AgCl as the reference, and a platinum wire as the counter electrode. The Ag/AgCl reference electrode was calibrated against a platinum gauze electrode in a hydrogen-saturated solution to obtain the RHE potential. The electrolyte for the HMFOR system was 1 M KOH containing 0.05 M HMF.

### Determination of the Products

For quantitative analysis of products, High performance liquid chromatography (HPLC, PerkinElmer LC-300 system, America) with an ultraviolet visible detector was used to analyze HMF oxidation products. The mobile phase A was methanol, and phase B was 5 mM ammonium formate aqueous solution, the volume ratio of A:B is 3:7, the flow rate is 0.6 mL min^−1^. For each analysis, 20 μL of the electrolyte after the potentiostatic electrolysis process was diluted to 4 mL with distilled H_2_SO_4_. Using a 4.6 mm × 150 mm Phenomenex Titank 5 μm C18 column.

HMF and oxidation products were quantitatively determined based on the calibration curves of those standard solutions. The performance metric parameters involving, HMF conversion, product yield, FDCA selectivity and Faradaic efficiency (FE) for the electrocatalytic oxidation of HMF were calculated by the following equations:

1 $${\text{HMF conversion }}(\% ) = \frac{{n ({\text{HMF}} \, {\text{consumed}})}}{{n ({\text{HMF}} \, {\text{initial}})}} \, \times \, 100\%$$

2 $${\text{Product yield}}\;(\% )=\frac{{n ({\text{product formed}})}}{{n ({\text{HMF}} \, {\text{initial}})}} \times 100\%$$

3 $${\text{FDCA selectivity}}\;(\%)=\frac{{n ({\text{FDCA}} {\text{formed}})}}{{n ({\text{total oxidative}} \, {\text{products}})}} \times \, 100\%$$

4 $${\text{FE}}(\% ) = \frac{{6 \cdot F\cdot n ({\text{FDCA}} {\text{formed}})}}{{{\text{total charge}} \, {\text{passed}} (C)}} \times \, 100\%$$

where F is the Faraday constant (96,485 C mol^−1^).

### Computational Methods

We have employed the first-principles tool-*Vienna *Ab initio* Simulation Package* (VASP) [28, 29] to perform all DFT calculations within the generalized gradient approximation (GGA) using the *Perdew-Burke-Ernzerhof* (PBE) [30] formulation. We have chosen the projected augmented wave (PAW) potentials [31, 32] to describe the ionic cores and take valence electrons into account using a plane wave basis set with a kinetic energy cutoff of 450 eV. Partial occupancies of the Kohn − Sham orbitals were allowed using the Gaussian smearing method and a width of 0.05 eV. For the optimization of both geometry and lattice size, the Brillouin zone integration was performed with 0.04 Å^−1^ *Γ*-centered *k*-point sampling [33]. The self-consistent calculations applied a convergence energy threshold of 10^−5^ eV. The equilibrium geometries and lattice constants were optimized with maximum stress on each atom within 0.02 eV Å^−1^. The 15 Å vacuum layer was normally added to the surface to eliminate the artificial interactions between periodic images. The weak interaction was described by DFT + D3 method using empirical correction in Grimme’s scheme [34, 35]. 15 Å vacuum was applied to both the upper and lower surfaces of the model to eliminate image interactions. Structural optimization was carried out with energy and force convergence criteria setting at 1.0 × 10^−5^ eV and 0.02 eV Å^−1^, respectively. Spin polarization method was adopted to describe the magnetic system. The adsorption energy was calculated as: *E*_ads_ = *E*(*adsorbent)—*E*(*)—E(adsorbent). *E*(*adsorbent), *E*(*) and E(adsorbent) represent the total energy of * adsorbent, * and adsorbent molecule, respectively.

## Results and Discussion

### Synthesis and Structure Characterization of Pd-NiCo_2_O_4_

**Fig. 1:**
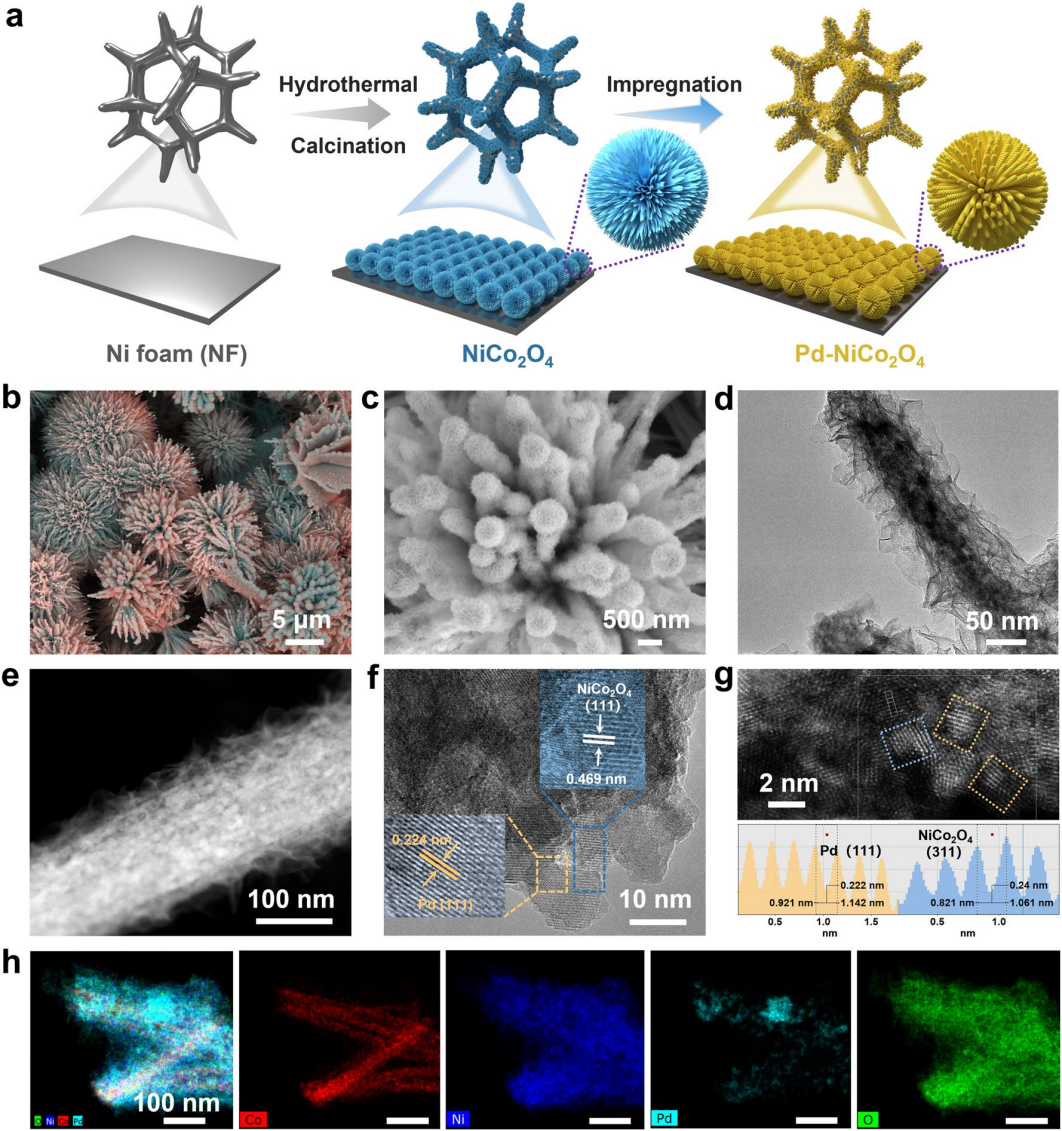
**a** Schematic diagram for preparation of Pd-NiCo_2_O_4_. **b, c** SEM images, **d** TEM, **e** STEM, **f** HR-TEM, **g** HAADF-STEM images and interplanar spacing profiles of Pd-NiCo_2_O_4_. **h** HAADF-STEM element mappings of Pd-NiCo_2_O_4_

The Co_3_O_4_ and NiCo_2_O_4_ on NF were synthesized by the same hydrothermal method and following calcination process. Pd-NiCo_2_O_4_ was obtained by impregnating the NiCo_2_O_4_ into a PdCl_2_ aqueous solution at 30 °C (Fig. 1a). Within 140 min, the color of aqueous solution gradually lightens, indicating the active component of Pd diffuses and adsorbs on NiCo_2_O_4_ surface. The morphologies of obtained electrocatalysts were observed by scanning electron microscopy (SEM) and TEM. The SEM images of Co_3_O_4_ exhibits a nanowire array structure (Fig. S1a-c). After Ni introduction, NiCo_2_O_4_ displays a three-dimensional (3D) blossom-cluster-like structure formed by the assembly of nanowires with smooth surfaces (Fig. S1d-f). After loading a small amount of Pd species onto NiCo_2_O_4_ (named Pd-NiCo_2_O_4_-1), the sample maintained the analogous blossom-cluster-like structure but formed by thicker, solid nanorods with rough surface and spherical top structure (Fig. S2a-c). With excessive Pd loading (named Pd–NiCo_2_O_4_-3), the catalysts demonstrated an accumulated micro-flower structure which assembled nanoplate (Fig. S2d-f). When loading an appropriate amount of Pd (Pd–NiCo_2_O_4_-2), the blossom-cluster-like is composed of columnar structure assembled by ultra-thin nanosheets, which grow outward from a common center (Fig. 1b, c). And this catalyst is represented by Pd–NiCo_2_O_4_ and is applied for subsequent characterization and testing. TEM images confirm the hierarchical columnar structure with ultra-thin nanosheets of Pd–NiCo_2_O_4_ (Fig. 1d, e). The unique configuration of structure is beneficial to expose active sites for promoted electrocatalytic performance. The high-magnification TEM (HR-TEM) and high-angle annular dark-field scanning transmission electron microscopy (HAADF-STEM) images illustrated that the Pd species possessed well-defined lattice fringes with an interplanar distance of 0.22 nm in the yellow regions, which was well matched to the (111) plane of the metallic Pd (Fig. 1f, g). The clear lattice fringes in blue regions with distances of 0.469 nm (Fig. 1f) and 0.24 nm (Fig. 1g) were indexed to the exposed NiCo_2_O_4_ (111) and (311) crystal planes, respectively.

Additionally, the element mapping images showed the Pd, Ni, and O elements distributed the whole nanocolumn and nanosheets homogeneously, while the Co element mainly concentrated in the inner nanocolumns (Fig. 1h). The surface atomic composition of Ni/Co was determined to be about 2:1 by energy dispersive X-ray spectroscopy (EDS), in which the atomic ratio of Ni to Co is discrepant to the feeding ratio of 1:2 (Fig. S3). The Pd, Ni, Co contents in bulk phase were determined to be 10.8, 32.94, and 19.54 wt% respectively by ICP analysis (Table S1). This abnormal Ni/Co ratio may be attributed to the partial dissolution of nickel foam substrate and its participation in the synthesis, as well as the predominant distribution of cobalt in the inner layers that was not detected.

**Fig. 2:**
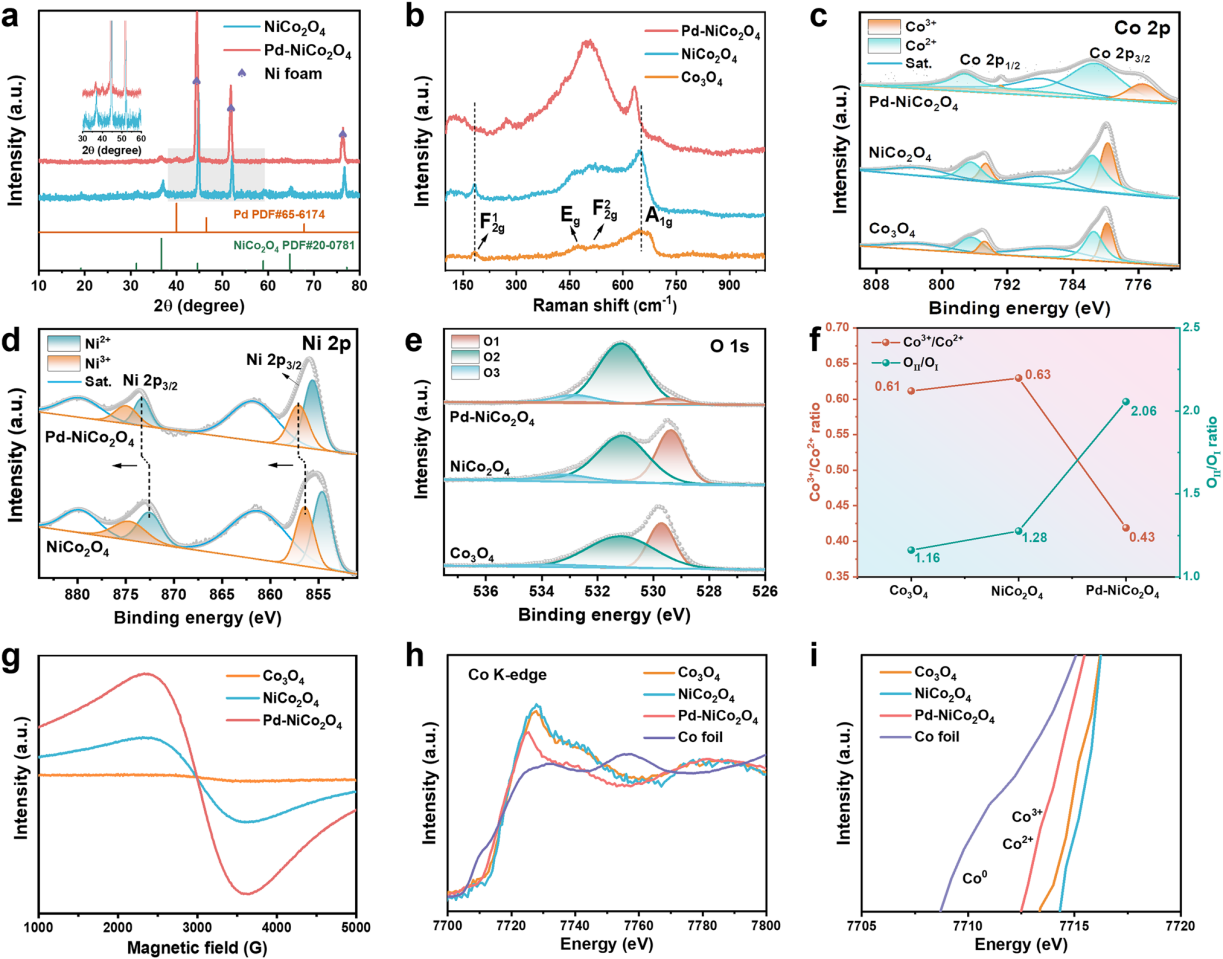
**a** XRD patterns of NiCo_2_O_4_, Pd-NiCo_2_O_4_. **b** Raman spectra of Co_3_O_4_, NiCo_2_O_4_, Pd-NiCo_2_O_4_. High-resolution **c** Co 2*p*, **d** Ni 2*p*, **e** O 1*s* XPS spectra of Co_3_O_4_, NiCo_2_O_4_, Pd-NiCo_2_O_4_. **f** The Co^3+^/Co^2+^ and O_II_/O_I_ ratios obtained from XPS spectra of samples. **g** EPR spectra and **h, i** Co K-edge XANES spectra of Co_3_O_4_, NiCo_2_O_4_, Pd-NiCo_2_O_4_

The crystal structure of catalysts was analyzed by XRD patterns results. The distinct peaks of Co_3_O_4_ and NiCo_2_O_4_ agreed well with PDF standard cards of Co_3_O_4_ (PDF#43–1003) and NiCo_2_O_4_ (PDF#20–0781), respectively (Fig. S4). The diffraction profiles of Co_3_O_4_ and NiCo_2_O_4_ are similar, attributed to the comparable cationic radius between Ni and Co, indicating that partial substitution of Ni does not alter the cubic spinel structure. Obviously, the XRD diffraction pattern of Pd-NiCo_2_O_4_-x remained peaks corresponding to NiCo_2_O_4_ and appeared new diffraction peaks at 40.1°, 46.6°, and 68.1°, indexing to the (111), (200), and (220) lattice planes of cubic Pd (PDF#65–6174), suggesting that the spinel structure of NiCo_2_O_4_ remained unchanged after Pd loading (Figs. 2a and S4b). Among different amount of Pd loading materials, Pd-NiCo_2_O_4_ exhibits the weakened and broadened diffraction peaks of Pd and NiCo_2_O_4_ phase (Fig. S4b), suggesting the possibility of increasing crystal defects in the material. In addition, the crystal structure of samples was investigated by Raman spectra. The characteristic signals of Co_3_O_4_ at 182 and 652 cm^−1^ assigned to asymmetric and symmetrical stretching vibration mode of oxygen atoms in tetrahedral coordination sites [CoO_4_] (F_2g_^1^) and octahedral coordination sites [CoO_6_] (A_1g_) (Fig. 2b), respectively [36, 37]. The signal at 471 and 522 cm^−1^ are attributed to vibration mode of O − Co − O bond of [Co^2+^]_Td_ (E_g_) and F_2g_^2^ models of spinel Co_3_O_4_ (Fig. 2b), respectively [38]. In comparison to Co_3_O_4_, the A_1g_ mode of NiCo_2_O_4_ showed slightly redshift, corresponding to the increase of the Co − O bond length [39, 40]. This indicates that the incorporated Ni sites mainly replace [CoO_6_] in the octahedral coordination. Regarding the F_2g_^1^ mode, compared with Co_3_O_4_, no obvious shift can be observed in NiCo_2_O_4_, suggesting the absence of Co − O bond deformation in tetrahedra, which further proving that Ni is inserted at the octahedral site. For NiCo_2_O_4_, the introduction of Ni will occupy the octahedral sites, while Co is distributed on the octahedral and tetrahedral coordination sites. The lattice distortion-induced redshift of the Raman peak of NiCo_2_O_4_ is conducive to the generation of oxygen vacancies [41]. However, the disappearance of the F_2g_^1^ mode signal was observed from Pd-NiCo_2_O_4_, which was attributed to the destruction of the long-range order of the crystal structure and an increase in oxygen vacancies following Pd loading [38, 39]. In addition, as a result of partially Ni substitution and Pd loading, lattice distortion or deficient coordination of oxygen causes a substantial downward shift of the highly symmetrical A_1g_ band [39].

The surface chemical valence states for all elements were investigated by XPS. The survey spectra showed the presence of Ni, Co, and O signals (Fig. S5a). The absence of Pd signal in the survey spectrum of Pd-NiCo_2_O_4_ is due to the low content of Pd (0.364 wt%) on the surface (Fig. S3, Table S2). In the high-resolution spectrum of Pd 3*d*, the XPS peaks located at 337.9 and 343.2 eV could be assigned to Pd 3*d*_3/2_ and 3*d*_5/2_, indicating that the main valence state of Pd was metal state (Fig. S5b) [42]. In the spectra of Co 2*p*, the peaks of Co_3_O_4_ and NiCo_2_O_4_ demonstrates a negligible change, both of which contain two characteristic spin-orbital doublets (Fig. 2c). The XPS peaks located at ≈779.88/794.78 eV and 781.68/796.58 eV are assigned to Co^3+^ and Co^2+^, respectively [43]. After loading Pd species onto the NiCo_2_O_4_, the peaks corresponding to Co^3+^ display an obvious negative shift to lower banding energy, suggesting that cobalt could be an electron acceptor in electron transfer between NiCo_2_O_4_ and Pd. The Ni spectrum of NiCo_2_O_4_ presented the coexistence of Ni^3+^ and Ni^2+^ with a Ni^3+^/Ni^2+^ ratio of 0.795, which was proved by the peaks located at 856.4/874.5 and 854.6/872.5 eV (Fig. 2d) [44]. The dominant Ni^2+^ for the Ni dopant suggested that Ni atoms replaced a tiny part of Co^2+^ in Co_3_O_4_, leading to the slight increase of Co^3+^/Co^2+^ ratio from 0.61 to 0.63 (Fig. 2f) [37, 45]. As for Pd-NiCo_2_O_4_, the peaks of both Ni^2+^ and Ni^3+^ shift to higher binding energy relative to NiCo_2_O_4_ (Table S3), suggesting the electronic interaction between Pd and the Ni [46, 47].

The O 1*s* spectra of samples can be deconvoluted into three sub-bands: O_I_, O_II_, and O_III_, which located at ≈529.3, 531.1, and 533.1 eV, respectively, corresponding in turn to lattice oxygen, coordinative oxygen vacancy and unavoidable surface adsorbed water molecules (Fig. 2e) [41, 48]. The introduction of Pd sites results in a decrease in the proportion of lattice oxygen, suggesting an enhanced presence of oxygen vacancies within the structure [49]. The ratio of O_II_/O_I_ is commonly utilized to assess the proportion of surface oxygen vacancies caused by defective oxidizing groups [37, 50]. From Fig. 2f, the calculated O_II_/O_I_ ratio form O 1*s* spectra shows that the Pd-NiCo_2_O_4_ possesses the maximum value, implying the highest concentration of surface oxygen vacancies (Vo). The existences of oxygen vacancies can be proved by electron paramagnetic resonance (EPR) spectra. The evident signals at g = 2.003 of NiCo_2_O_4_ and Pd–NiCo_2_O_4_ clearly indicate the presence of abundant oxygen vacancies in both materials (Fig. 2g) [51], which is consistent with the findings from Raman and O 1*s* XPS spectra. Furthermore, the intensified EPR signals observed on Pd–NiCo_2_O_4_ suggest higher concentrations of oxygen vacancies, thereby explaining why Pd-NiCo_2_O_4_ exhibits lower Co^3+^/Co^2+^ and Ni^3+^/Ni^2+^ ratios compared to those of NiCo_2_O_4_ (Figs. 2f and S6). To gain deeper insights into the electronic configuration of the samples, X-ray absorption near-edge structure (XANES) analysis of the Co-K-edge was conducted (Fig. 2h). In comparison to the Co foil reference (Co^0^), all three of Co_3_O_4_, NiCo_2_O_4_ and Pd-NiCo_2_O_4_ exhibit absorption edges shifting towards higher energy levels, suggesting an elevation in their respective Co valence states (Fig. 2i). Conversely, the K-side absorption energy of Ni in Pd–NiCo_2_O_4_ is greater than that of NiCo_2_O_4_, indicating a change in electron density around Ni (Fig. S7). The electronic properties of the transition elements obtained from the XANES are highly consistent with the surface chemistry, which is in good agreement with the discovery of XPS.

### Electrocatalytic Performances and Product Analysis for HMFOR

**Fig. 3:**
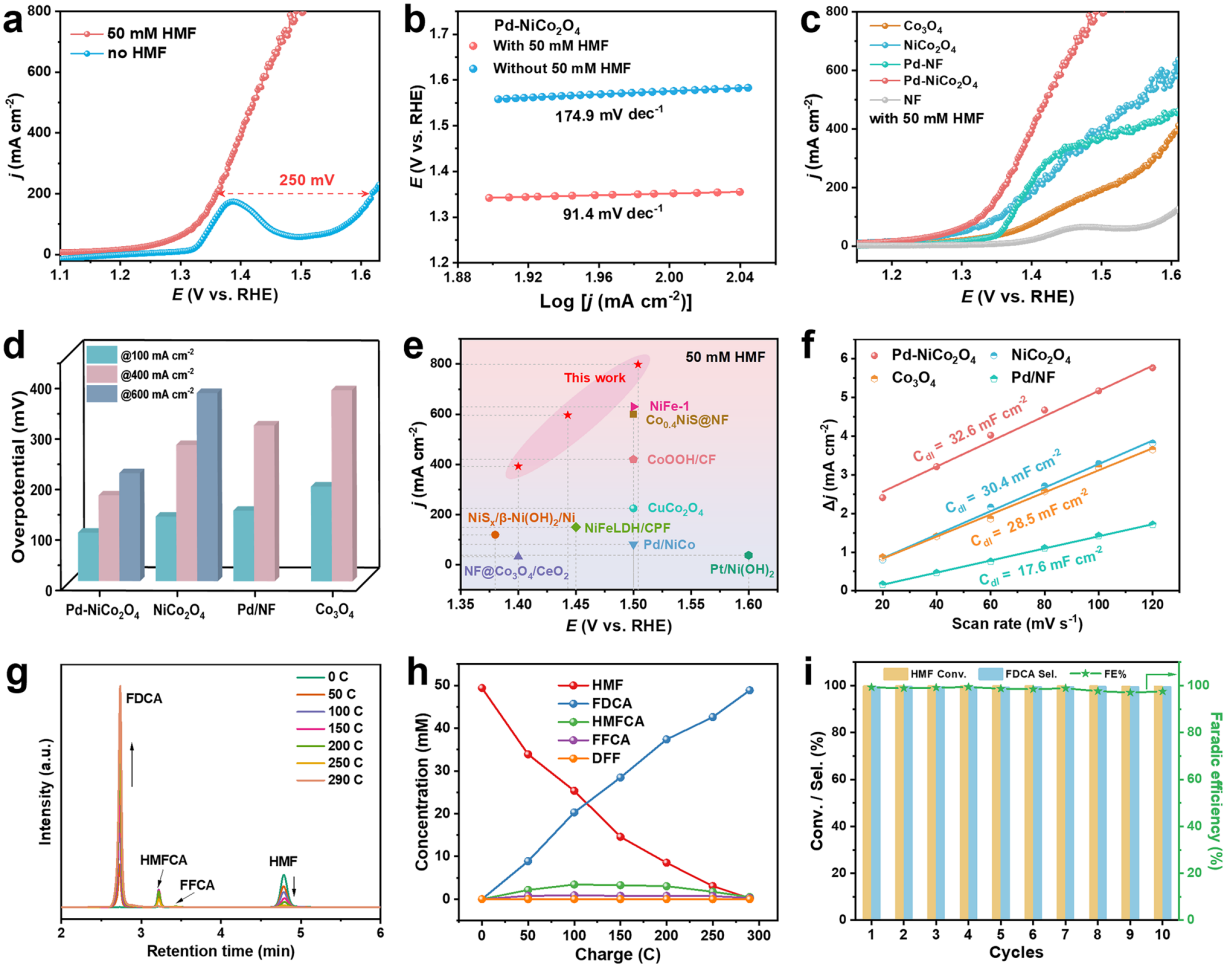
**a** LSV curves and **b** Tafel slope of Pd-NiCo_2_O_4_ in 1.0 M KOH with and without 50 mM HMF. **c** LSV curves of different samples in 1.0 M KOH with 50 mM HMF. **d** Overpotential of the samples at various current densities for HMFOR. **e** Comparison of Pd-NiCo_2_O_4_ performance with reported HMF oxidation catalyst in 50 mM HMF. **f** *C*_dl_ values of the samples. **g** HPLC chromatogram, and **h** the concentration changes of oxidation products during HMFOR at 1.5 V. **i** FE, selectivity of FDCA, and the conversion of HMF obtained by the Pd-NiCo_2_O_4_ over ten consecutive cycles of HMFOR

The electrocatalytic performance of the as-synthesized materials was evaluated in a standard three-electrode system. First, linear sweep voltammetry (LSV) curves were conducted the to assess electrocatalytic activity of samples in 1 M KOH with 50 mM HMF. Visibly, as for Pd-NiCo_2_O_4_-x (x = 1, 2, 3), with the increase in Pd introduction (corresponds to x from 1 to 3), a trend was observed in which the oxidation current density exhibited both an increase and subsequent decrease (Fig. S9). Similarly, the propensity current density of Pd-NiCo_2_O_4_-x can be observed in 1 M KOH with 100 mM HMF, suggesting the presence of an optimal Pd loading content when x = 2 (Fig. S8). Unless otherwise stated, Pd-NiCo_2_O_4_-2 denoted as Pd-NiCo_2_O_4_ in the in the full text. In Fig. 3a, the LSV curves in 1 M KOH with or without 50 mM HMF of the Pd–NiCo_2_O_4_ electrode were compared. The Pd–NiCo_2_O_4_ required an onset potential (defined here as the potential at 5 mA cm^−2^) of 1.26 V versus RHE for OER. Upon the addition of HMF, the onset potential dramatically decreased to 1.0 V versus RHE, indicating that the oxidation of HMF at the Pd–NiCo_2_O_4_ electrode is thermodynamically more favorable than OER. The Tafel slope of the HMFOR (91.4 mV dec^−1^) is lower than that of OER (174.9 mV dec^−1^), revealing faster electron transfer and reaction kinetics between the interface of HMF and Pd–NiCo_2_O_4_ electrocatalysts (Fig. 3b). The HMFOR performance of different electrodes is shown in Fig. 3c. It is evident that Pd–NiCo_2_O_4_ achieved an industrial current density of 800 mA cm^−2^ at 1.5 V versus RHE in 1 M KOH with 50 mM HMF. The disparity in activity between NiCo_2_O_4_/Pd–NiCo_2_O_4_ and Co_3_O_4_/Pd–Co_3_O_4_ further underscores the benefits of electronic structure modification by cationic substitution in HMFOR catalysis. Besides, the Pd/NF displays comparable HMFOR performance to NiCo_2_O_4_, and Pd–Co_3_O_4_ exhibits considerable early potential than Co_3_O_4_, indicating that Pd loading plays a crucial role in HMFOR (Figs. 3c and S10). Additionally, among the samples, Pd–NiCo_2_O_4_ electrode showed the lowest overpotential for HMFOR at different current densities in 1 M KOH with both 50 and 100 mM HMF (Figs. 3d and S11), demonstrating that the Pd-NiCo_2_O_4_ electrode exhibits faster reaction kinetics and higher reaction activity toward the HMFOR. This outstanding HMFOR activity exceeds that of state-of-the-art metal oxides incorporating noble metals and other representative catalysts reported thus far (Fig. 3e, Table S4). Meanwhile, we further investigated the electro-oxidation performance of furfural (FF) and furfuryl alcohol (FA) on the Pd–NiCo_2_O_4_ and NiCo_2_O_4_ electrodes, and there is a significant performance enhancement observed from the Pd–NiCo_2_O_4_ electrode (Fig. S12), indicating that the presence of Pd heteroatoms can universally promote the oxidation of alcohol (R-OH) and aldehyde (R-CHO) groups in alkaline solutions.

In cases where the reactions at the electrode interface are primarily limited by charge transfer rather than mass transfer diffusion, the electrochemical impedance spectroscopy (EIS) technique typically exhibits a semicircular response [52]. The Nyquist plot of Pd–NiCo_2_O_4_ in 1 M KOH demonstrates a semicircle with the smallest diameter compared to NiCo_2_O_4_ and Co_3_O_4_ (Fig. S13), proving that faster charge transfer can be achieved on the surface of the electrocatalysts via Ni substitution and Pd loading. In addition, the electrochemical surface area (ECSA) was investigated by evaluating the electrochemical double layer capacitance (*C*_dl_) in the non-faradaic regions using cyclic voltammetry (Fig. S14). As demonstrated in Fig. 3f, Pd–NiCo_2_O_4_ shows a larger C_dl_ than those of NiCo_2_O_4_, Co_3_O_4_ and Pd/NF, signifying that the blossom-cluster-like with ultra-thin nanosheets is beneficial to expose active sites for promoted electrocatalytic performance. According to the proportional relationship ECSA and C_dl_ (ECSA = C_dl_/C_s,_ C_s_ = 0.04 mF cm^−2^), the ECSA of Pd-NiCo_2_O_4_, NiCo_2_O_4_, Co_3_O_4_, and Pd/NF is 852.5, 760, 712.5, and 390, respectively. To evaluate the intrinsic activity of electrocatalysts, the LSV curves with ECSA normalization were collected of the samples (Fig. S15), where Pd–NiCo_2_O_4_ exhibits superior normalized current density than NiCo_2_O_4_, Co_3_O_4_. And the Pd/NF displays a slightly lower intrinsic activity than Pd–NiCo_2_O_4_, indicating that the introduction of Pd and Ni synergistically increased the intrinsic HMFOR activity on Pd–NiCo_2_O_4_ electrode. The HER performances of samples were also investigated in 1 M KOH solution. Apparently, among the obtained samples, Pd–NiCo_2_O_4_ exhibits the best HER activity, which requires a potential of 88 mV to achieve −20 mA cm^–2^ (Fig. S16a). Besides, Pd–NiCo_2_O_4_ displays weak current fluctuations during continuous chronopotentiometry test for over 48 h at – 150 mA cm^–2^, manifesting the superior robust performance (Fig. S16b).

To detect the oxidation products after I-t electrocatalysis, high-performance liquid chromatography (HPLC) was conducted (Fig. S17). As exhibited in Fig. 3g, as the charge accumulates, the peak intensity of HMF gradually decreases, while that of FDCA gradually increases. The intermediate of HMFCA are gradually consumed, with almost no 2,5-diformylfuran (DFF) signal peak. When the transfer charge reaches 289 C (theoretical charge), the concentration of HMF decreased to zero and the concentration of FDCA is maximized (Fig. 3h). These results indicate that the HMF oxidation on Pd-NiCo_2_O_4_ proceeded path of HMF → HMFCA → FFCA → FDCA. Furthermore, the selectivity of FDCA and Faradaic Efficiency (FE) at different potentials of Pd-NiCo_2_O_4_ (Fig. S18) and contrast samples are quantified (Fig. S19). As expected, Pd-NiCo_2_O_4_ exhibited superior FDCA selectivity (99.2%) and FE (99.6%) comparison NiCo_2_O_4_ and Co_3_O_4_ at 1.5 V versus RHE (Fig. S19). Above all, the Pd-NiCo_2_O_4_ electrode retains negligible decline of HMF conversion, FDCA selectivity and FE over 10 consecutive cycles (Fig. 3i), suggesting the robust stability of Pd-NiCo_2_O_4_ for promising practical HMFOR application.

After HMFOR test, a series characterization was employed to assess the stability of structure and composition. SEM and TEM images indicate that the general outline of 3D blossom-cluster-like structure of Pd–NiCo_2_O_4_ remains well, and only the nanosheets assembled into nano-column are more compact (Fig. S20a-d). Additionally, the interplanar spacings of 0.243 and 0.223 nm corresponding to NiCo_2_O_4_ (111) and Pd (111) planes can be identified respectively (Fig. S20e, f). SEM, TEM, and HR-TEM combined with an evenly dispersed distribution of Pd, Ni, Co, and O elements (Fig. S20g) results manifest the stable microstructure and crystal structure of Pd–NiCo_2_O_4_. The surface state of elements was also investigated by XPS analyzation. Different from initial Pd–NiCo_2_O_4_, the spectrum peak of Pd can be clearly recognized form the XPS survey spectra after HMFOR process (Fig. S21), implying more Pd clusters are exposed on the Pd–NiCo_2_O_4_ surface. Slight alterations in the high-resolution XPS spectra of Pd, Co, and Ni indicate a subtle modification in the surface chemical state of Pd–NiCo_2_O_4_ (Fig. S22). All those analyses prove the preeminent robustness of Pd–NiCo_2_O_4_ in HMF oxidation.

### Experimental Analysis of HMF Oxidation Mechanism

**Fig. 4:**
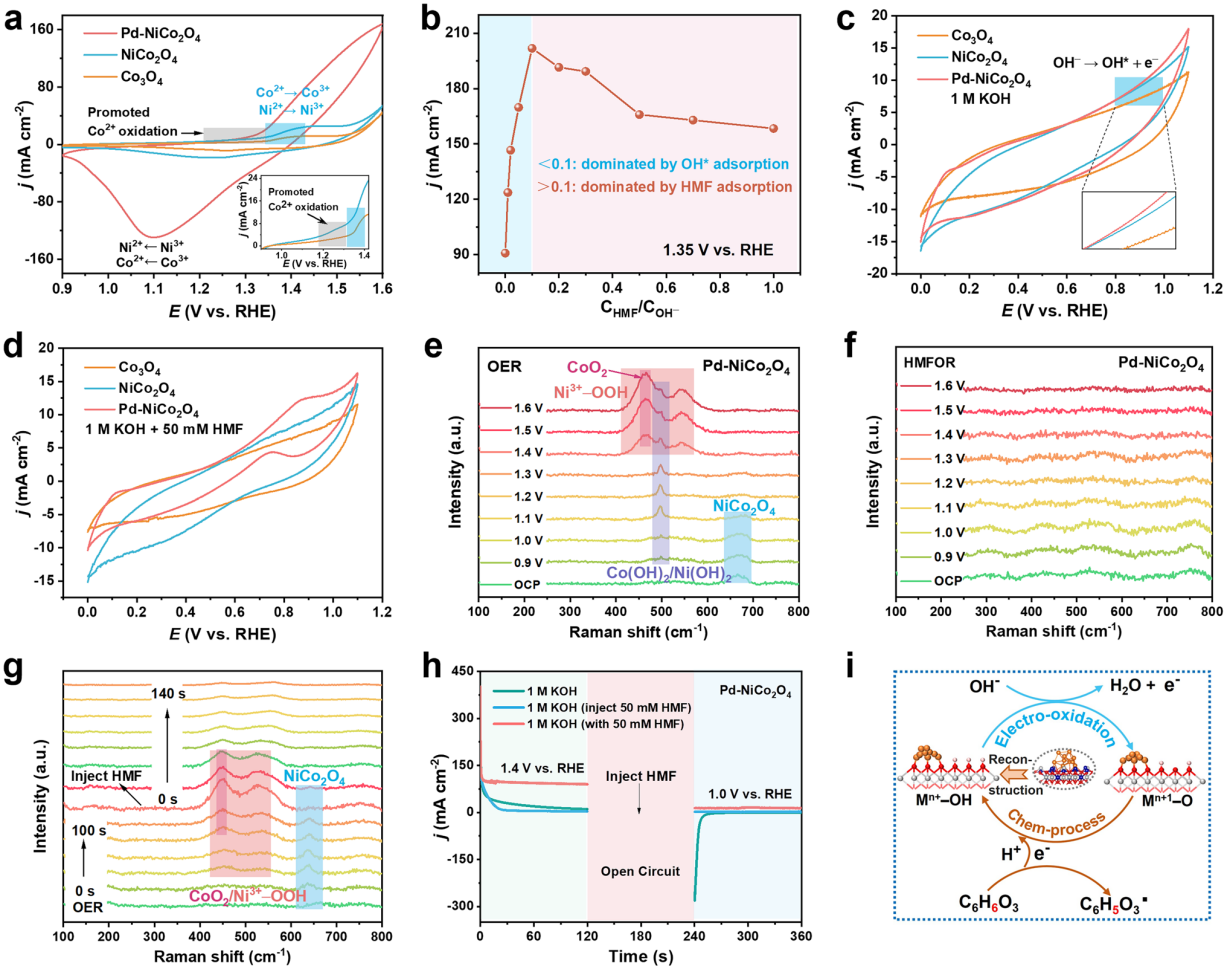
**a** CV curves of Co_3_O_4_, NiCo_2_O_4_ and Pd-NiCo_2_O_4_ in 1 M KOH. **b** Relationship between the current density and ratio of C_HMF_/C_OH_^–^ at 1.35 V_RHE_. CVs of Co_3_O_4_, NiCo_2_O_4_ and Pd-NiCo_2_O_4_ in Ar-saturated **c** 1.0 M KOH and **d** 1.0 M KOH with 50 mM HMF at a scan rate of 50 mV s.^−1^. In situ Raman spectra of Pd-NiCo_2_O_4_ in **e** 1 M KOH, **f** 1 M KOH with 50 mM HMF and **g** half-way injected HMF. **h** Multipotential-step curves of Pd-NiCo_2_O_4_ in 1 M KOH solution with and without 50 mM HMF addition. **i** Scheme of the indirect mechanism for HMFOR on Pd-NiCo_2_O_4_

To gain a comprehensive and deep insights to the electrocatalytic HMFOR mechanism, electrochemical measurement technologies and in situ Raman spectroscopy were performed in 1 M KOH with or without HMF. As shown in Fig. 4a, the oxidation current ranges from 1.35 to 1.4 V versus RHE in 1 M KOH can be assigned to Ni^2+^/Co^2+^ oxidation to active Ni^3+^/Co^3+^ species. In addition, the NiCo_2_O_4_ exhibits the relatively lager anodic peak at ranges from 1.2 to 1.35 V versus RHE than that of Co_3_O_4_, suggesting the promoting effect of Ni incorporation on Co^2+^ pre-oxidation [53]. After introducing 50 mM HMF, the anodic current density of NiCo_2_O_4_ and Co_3_O_4_ exhibited a rapid increase together with the oxidation of Co^2+^, and the corresponding redox couples vanished, suggesting the involvement of Co oxidation in HMFOR (Fig. S23). On Pd–NiCo_2_O_4_ electrode, OER occurs overlaps with Ni^2+^ oxidation peaks and becomes overwhelming at about 1.4 V versus RHE, revealing the dominant role of Pd in OER [18]. It can be reasonably speculated that Pd is conducive to promoting the formation of highly active Ni^3+^ species, which is beneficial to indirect oxidation pathway. An increase in the oxidation current density can be observed in the voltage range below 1.35 V versus RHE (Fig. S24), indicating that potential-dependent oxidation (direct oxidation) of HMF occurred in this potential range.

As noted above, the direct oxidation mechanism, involving competitive adsorption of OH^−^ and react substances, is significant for HMFOR performance on Pd–NiCo_2_O_4_ electrode. Therefore, the relationship between the current density of HMFOR and the concentration ratio of adsorbed HMF and KOH (C_HMF_/C_OH_^−^) were studied (Fig. S25). As shown in Fig. 4b, the result exhibits a volcano-shaped curve with an optimal C_HMF_/C_OH_^−^ ratio of 0.1 for achieving the maximum current density. The anode current response increased with the increase of HMF concentration until the HMF concentration is 100 mM. The concentration dependence of current density indicates that the competitive adsorption of OH* and HMF leads to the direct oxidation of HMFOR on Pd–NiCo_2_O_4_ electrode. As the concentration of HMF continued to increase (C_HMF_/C_OH_^−^ > 0.1), the electrode surface is dominated by adsorbed OH*. Once HMF molecules are adsorbed onto the electrode, they are immediately oxidized in regions of enriched OH* species. Consequently, the current density is positively correlated with C_HMF_/C_OH_^−^ ratio. When the concentration ratio exceeds 0.1, the electrode surface is predominantly occupied by adsorbed HMF molecules. And there are not enough OH* species to react with excessive HMF, resulting in a decrease in current density with the increase of HMF concentration (Fig. S26).

Furthermore, CV tests in Ar-saturated 1 M KOH solution were conducted to investigate the adsorption behavior of OH* on Pd–NiCo_2_O_4_, NiCo_2_O_4_ and Co_3_O_4_ electrodes. In the potential range of 0.8–1.05 V versus RHE, there is a distinct oxidation current, which is attributed OH^−^ oxidation to OH* (OH^*−*^ → OH + e^*−*^), corresponding to the OH* adsorption region (Fig. 4c) [54–56]. Regardless of the addition of HMF, both Pd–NiCo_2_O_4_ and NiCo_2_O_4_ exhibit more negative OH* onset potentials and higher oxidation currents than Co_3_O_4_ (Fig. 4c, d). It is noteworthy that in 1 M KOH solution, the oxidation current of NiCo_2_O_4_ is significantly higher than that of Co_3_O_4_, but comparable to the oxidation current of Pd–NiCo_2_O_4_, indicating that the Ni incorporation significantly promotes the adsorption of OH*. In situ attenuated total reflectance Fourier transform infrared spectroscopy (ATR-FTIR) was used to further evaluate the adsorption behavior of HMF and OH^−^. As shown in Fig. S27, the higher O–H vibration peaks (2700–3800 cm^−1^) intensity indicate that the adsorption of OH^−^ by NiCo_2_O_4_ is stronger than that of Pd–NiCo_2_O_4_. On the contrary, Pd–NiCo_2_O_4_ shows a stronger C=O stretching peak (~ 1600 cm^−1^) than NiCo_2_O_4_, indicating a stronger adsorption of HMF.

To further investigate the oxidation mechanism of Pd–NiCo_2_O_4_, in situ Raman spectra were conducted. For OER process (Fig. 4e), at open circuit potential (OCP), the Raman spectrum displays a characteristic peak at 667 cm^−1^ associated with NiCo_2_O_4_. As the voltage is increased, the intensity of this peak gradually diminishes and eventually disappears at 1.4 V versus RHE, indicating the transformation of NiCo_2_O_4_. Throughout the electrolysis process (0.9–1.6 V versus RHE), the Raman band at 496 cm^−1^ is attributed to structural defects or A_1g_ stretching modes of Ni^2+^–OH/Co^2+^–OH [57]. This band reaches maximum intensity at 1.3 V versus RHE before weakening, suggesting that applied voltage drives the Pd-NiCo_2_O_4_ to undergo surface reconfiguration [58]. Upon surpassing a voltage of 1.35 V versus RHE, two distinctive Raman signals at 465 and 542 cm^–1^ emerge in the spectrum, corresponding to the E_g_ bending and A_1g_ stretching vibrational modes of Co^3+^–O/Ni^3+^–O bond in CoO_2_/Ni^3+^–OOH [59]. This observation suggests the surface construction generates active high-valent NiCo (oxy) hydroxides species on Pd-NiCo_2_O_4_. In the HMFOR process, no characteristic peaks corresponding to Co^3+^–O/Ni^3+^–O bonds were observed after 1.1 V versus RHE, indicating a dynamic transformation between Co^3+^–O/Ni^3+^–O phase and HMF (Fig. 4f). To elucidate the reasons for the inconsistent results, HMF was injected midway through the OER electrolysis process (Fig. 4g). During the initial 100 s of the OER process, with the emergence and gradual growth of Co^3+^–O/Ni^3+^–O peaks, the characteristic peaks associated with NiCo_2_O_4_ gradually disappeared. Following the injection of HMF, the peak corresponding to Co^3+^–O/Ni^3+^–O gradually diminished, implying the oxidized NiCo (oxy)hydroxides species are the real active phase of HMFOR. Due to the near absence of detection of high-valent Co^3+^–O/Ni^3+^–O in Fig. 4f, it is suggested that high oxidation state NiCo (oxy)hydroxides have a rapid redox reaction with HMF, and the consumption rate exceeds the electrooxidation generation rate, resulting in undetectable accumulation on the electrode surface. The indirect oxidation mechanism was further investigated via multipotential-step curve measurements. From Fig. 4h, the electrode is electrooxidized to enrich high-valent substances of NiCo (oxy) hydroxides at a higher potential (1.45 V versus RHE). Upon lowering the potential to 1.0 V versus RHE, the reduction current of the high oxidation state (oxy)hydroxides can be observed at 1 M KOH, indicating that the initially formed hydroxides can accumulate and remain stable during the OCP stage. However, no significant negative current can be observed in either the initial or midway injection of HMF, suggesting the absence of (oxy) hydroxides accumulation, which is consistent with in situ Raman results. A similar phenomenon can be observed on NiCo_2_O_4_ and Co_3_O_4_ electrodes (Fig. S28). This can be attributed to the active species generated by electrooxidation will be consumed instantaneously by the spontaneous oxidation reaction with HMF, preventing the accumulation of high-valent (oxy)hydroxides on the electrode surface, which is consistent with existing literature reports [37, 60]. Thus, it is reasonable to conclude that the Pd–NiCo_2_O_4_ catalyze HMFOR also occurs through an indirect oxidation mechanism (electrochemical-chemical (E–C) pathway). Specifically, the electrodes undergo an electrically driven surface reconstruction process to form hydroxides and a subsequent deprotonation process to generate high-valent (oxy) hydroxides. The electro-generated active NiCo (oxy) hydroxides can rapidly and spontaneously capture protons and electrons from HMF through a chemical process. Consequently, the HMF molecules are spontaneous oxidized while high-valent NiCo (oxy)hydroxides are chemical reduced to Ni^2+^–OH/Co^2+^–OH (Fig. 4h). Therefore, the E-C oxidation pathway and OH*-participated oxidation pathway jointly determines the HMF oxidation performance on the electrode.

### Density Functional Theory Simulation

**Fig. 5:**
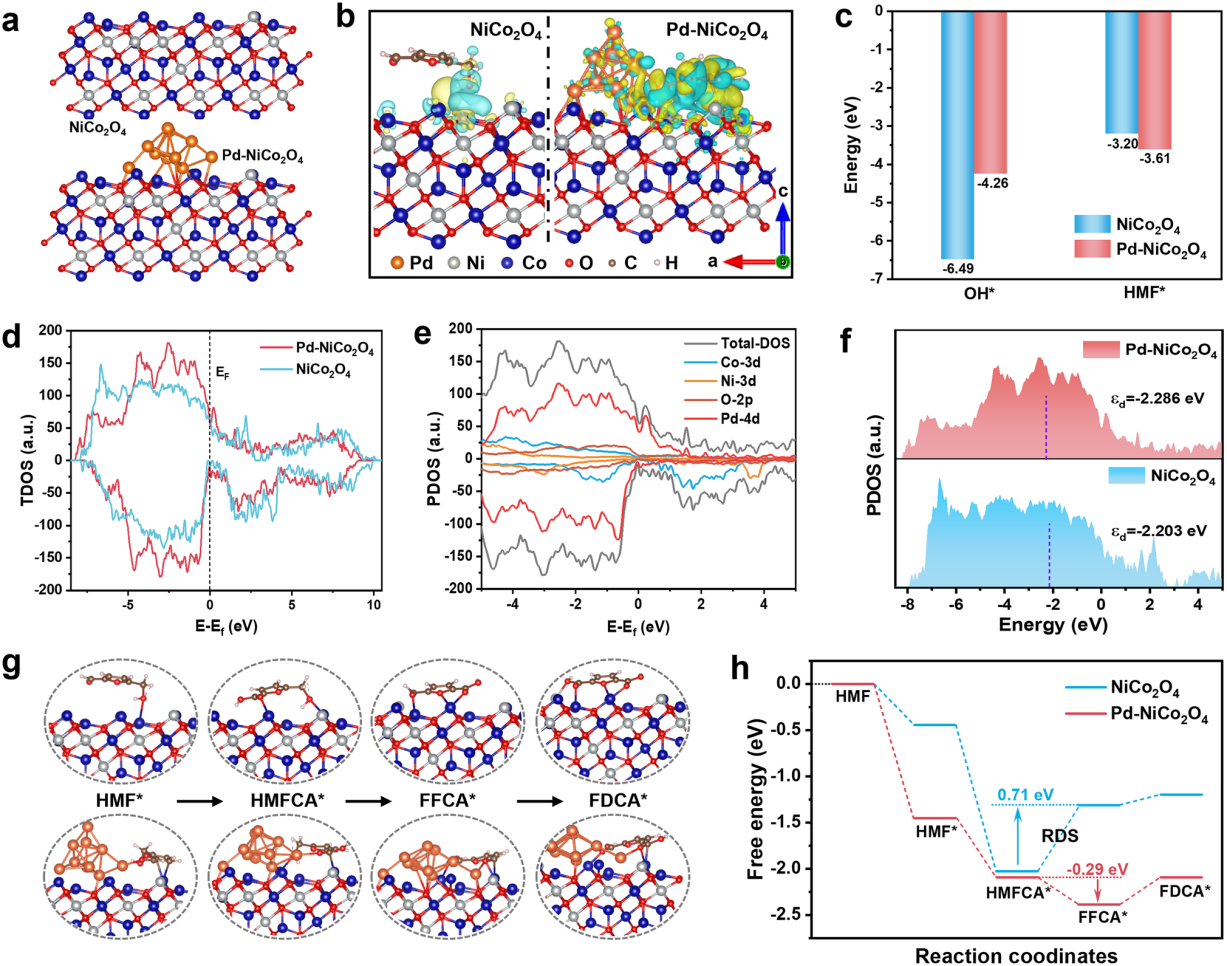
**a** DFT-optimized structures of NiCo_2_O_4_ and Pd-NiCo_2_O_4_. **b** Charge density difference for HMF adsorbing on the NiCo_2_O_4_ and Pd-NiCo_2_O_4_ (the yellow and cyan colors indicate charge accumulation and depletion, respectively). **c** HMF* and OH* adsorption energy on NiCo_2_O_4_ and Pd-NiCo_2_O_4_. **d** TDOS of NiCo_2_O_4_ and Pd-NiCo_2_O_4_. **e** PDOS of Pd-NiCo_2_O_4_. **f** d-Band centers of NiCo_2_O_4_ and Pd-NiCo_2_O_4_. **g** Optimized stable adsorption configurations for HMFOR and **h** corresponding Free energy diagram on NiCo_2_O_4_ and Pd-NiCo_2_O_4_

To further unravel the inner promoting mechanism of the integration of Pd on NiCo_2_O_4_ in HMFOR, DFT calculations were conducted. The optimized stable structures of NiCo_2_O_4_ and Pd-NiCo_2_O_4_ are presented in Fig. 5a, in which the (111) face of catalyst samples was selected to calculate. From the polarized local electronic configuration results, Pd introduction could cause local charge accumulations around neighboring Co atoms and electron loss around Ni (Fig. S29), which is consistence with the XPS results. After adsorption of HMF, Pd–NiCo_2_O_4_ transfers more electrons to HMF, suggesting a stronger interaction with HMF (Fig. 5b). Considering the proper adsorption behavior of OH^−^ and HMF is fundamentally essential for the HMFOR performance, the bonding strengths of OH* and HMF* on samples were evaluated. As shown in Fig. 5c, the Pd–NiCo_2_O_4_ (− 3.61 eV) exhibits more negative adsorption energies than that of NiCo_2_O_4_ (− 3.20 eV), indicating enhanced HMF adsorption behavior on Pd–NiCo_2_O_4_. While for OH* adsorption, the bonding strengths weaken form − 6.49 eV (NiCo_2_O_4_) to − 4.26 eV (Pd–NiCo_2_O_4_), suggesting that the loading of Pd with NiCo_2_O_4_ can moderate adsorption energy to balance the competitive adsorption of HMF molecules and OH^−^ species. And the optimized adsorption energy is beneficial to the desorption in subsequent steps [61, 62]. In addition, the introduction of Pd results in a total density of states (TDOS) near the Fermi level (E_F_) for Pd–NiCo_2_O_4_ higher than that of NiCo_2_O_4_ (Fig. 5d), indicating an enhancement in the conductivity of Pd–NiCo_2_O_4_, which is conducive to rapid electron transfer in HMFOR process. Partial density of states (PDOS) analyses reveals that Pd-4d orbital provides a major contribution to TDOS and shows strong peaks in proximity to the E_F_, implying that Pd acts as active site to boost electron transfer with low energy barriers (Figs. 5e and S30). As shown in Fig. 5f, the d-band center (ɛ_d_) of NiCo_2_O_4_ (− 2.203 eV) demonstrates a slight downshifts to − 2.286 eV after Pd introduction, implying weaker interaction with adsorbed oxidized intermediates [63], consisting with the weakened OH* adsorption behavior.

The reaction states of HMFOR processes on samples were then investigated. Figure 5g shows the specific adsorption configurations corresponding to the path of lowest energy. For NiCo_2_O_4_ sample, the hydroxyl group in HMF is mainly adsorbed at the Co site, and then the aldehyde group is adsorbed with H_2_O addition and dehydrogenation to HMFCA* (Fig. 5g). After the integration of Pd on the surface of NiCo_2_O_4_, the C=C group of the furan ring is mainly adsorbed at the Co site, while the aldehyde group of HMF is first adsorbed at the Pd site and then dehydrogenated to *HMFCA, and then *HMFCA is dehydrogenated to FFCA* and FDCA* between NiCo_2_O_4_ and Pd (Figs. 5g and S31). This indicates that the C=C group of the furan ring and aldehyde/hydroxyl group are mainly adsorbed at the Co and Pd sites, respectively. The reaction state of HMFOR further indicates that the dehydrogenation of HMFCA to FFCA is the rate-determining step (RDS) of NiCo_2_O_4_, and the reaction barrier is 0.71 eV, which is greatly higher than that of Pd-NiCo_2_O_4_ (− 0.29 eV) (Fig. 5h). This may be caused by the filling of OH^−^ into the oxygen vacancy (Vo) by a lattice oxygen oxidation process prior to coupling with HMF, thus accelerating the RDS of HMFCA intermediate dehydrogenation [64]. However, the RDS over Pd–NiCo_2_O_4_ is FFCA* → FDCA*. This can be attributed that the introduction of Pd lowers the adsorption energy of OH*, weakening the binding between intermediates and OH*. And Pd–NiCo_2_O_4_ is conducive to the adsorption of C=O, hindering desorption of FFCA* and forming the RDS of FFCA* → FDCA* [65].The results show that the cooperation of Pd on NiCo_2_O_4_ is conducive to accelerating the RDS and reaction conversion rate during HMFOR process, thereby promoting the enhancement of product selectivity and yield.

### Couple Reaction Performances

**Fig. 6:**
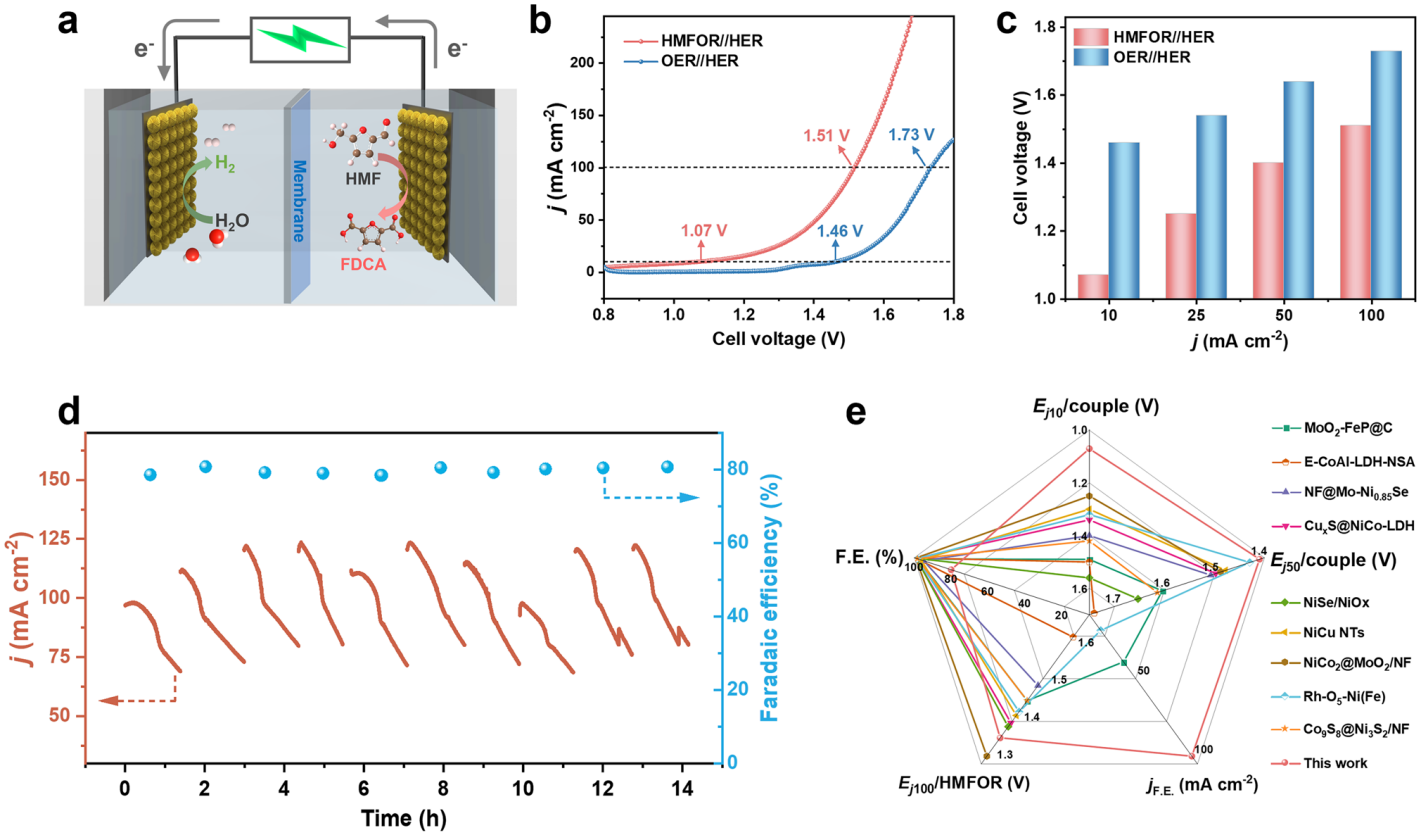
**a** Schematic illustration of coupled electrolysis HMFOR and HER. **b** LSV curves of Pd-NiCo_2_O_4_ for water splitting and hybrid coupling HMFOR with HER. **c** Comparison of the voltages required to achieve different current densities. **d** Stability test toward HMF oxidation to FDCA under ten successive electrolysis operations. **e** Comparison of the electrocatalytic performance of our catalyst and the recently reported advanced electrocatalysts

To assess the bifunctional activity and stability of the Pd–NiCo_2_O_4_ electrode, an integrated HMFOR coupled with HER (HMFOR//HER) dual-electrode electrolyzer was established (Fig. 6a). In comparison with overall water splitting (OER//HER), the hybrid coupling electrolysis demonstrates significantly lower cell voltage (Fig. 6b). At cell voltages as low as 1.02 and 1.4 V, current densities of 10 and 50 mA cm^–2^ could be respectively delivered, which are substantially lower than the cell voltages required for OER as the anodic reaction (Fig. 6c). To evaluate the electrolysis efficiency of the coupled two-electrode system, electrolysis at different voltages and product detection were performed (Fig. S32). Although relatively good Faradaic efficiency and yield of FDCA can be acquire at 2 V, to avoid carbon imbalance caused by excessive long-term electrolysis of HMF to produce humus, the cyclic electrolysis tests under high current density were conducted. From Fig. 6d, the continuous output current density of the electrolyzer could be stably maintained at 100 mA cm^–2^ for ten successive electrolysis. Under the influence of competitive OER, about 80% Faradaic efficiency is maintained for FDCA. It is noteworthy that the decline in current density during each electrolysis period and long-term test was due to the consumption of HMF (Figs. 6d and S33). Compared to catalysts reported in literatures, the synthesized Pd–NiCo_2_O_4_ catalyst exhibited exceptional performance in terms of cell voltage and high operation current density (Fig. 6e, Table S5), highlighting the promising prospects for integrating biomass upgrading and sustainable hydrogen production applications.

## Conclusions

In summary, a Pd–NiCo_2_O_4_ electrocatalyst system has been established by incorporating Ni into Co_3_O_4_ lattice to form NiCo_2_O_4_ and integrating Pd clusters on NiCo_2_O_4_ surface for biomass upgrading. Due to the Ni doping and Pd loading, the as-prepared Pd–NiCo_2_O_4_ exhibits an elevated industrial-level current density of 800 mA cm^−2^ at a potential of 1.5 V, as well as a high FE (99.6%) and FDCA yield (99.5%). And the catalyst can keep negligible decline of FE, selectivity, and product yield over 99% after running 10 continuous electrocatalytic cycles. Additionally, Pd–NiCo_2_O_4_ demonstrates remarkable coupling performance, achieving a low cell voltage of 1.51 V at 100 mA cm^−2^ and displaying operational stability during the HMFOR-assisted H_2_ production. Experiment results demonstrate that the doping of Ni promoted the generation of active Co^3+^–O and the oxidation of OH^−^ to electron-deficient OH*, boosting the direct oxidation process. Theoretical studies elucidate that the involvement of Pd on NiCo_2_O_4_ surface optimized the adsorption behavior of HMF and OH^−^ species by modulating the local electronic configuration of Ni/Co. These synergic improvements promote the electron/charge-transfer activities for HMFOR, promoting the key step of HMFCA deprotonation to FFCA. This study proposes a new-concept on the construction of high-performance electrocatalysts for biomass-upgraded coupling with hydrogen production from integrated system.

## Supplementary Information

Below is the link to the electronic supplementary material.

Supplementary file1 (DOCX 4742 KB)
