# Supplementary material for: Enhancing the Electrocatalytic Oxidation of 5-Hydroxymethylfurfural Through Cascade Structure Tuning for Highly Stable Biomass Upgrading
Source: Nanomicro Lett. 2024 Aug 22;16:275. doi: 10.1007/s40820-024-01493-3 (PMC11339012; doi:10.1007/s40820-024-01493-3)
Supplement: Supplementary file 1 — Supplementary file1 (DOCX 4742 KB) [file 40820_2024_1493_MOESM1_ESM.docx]

Supporting Information for

Enhancing the Electrocatalytic Oxidation of 5-Hydroxymethylfurfural through Cascade Structure Tuning for Highly Stable Biomass Upgrading

Xiaoli Jiang^1^, Xianhui Ma^2^, Yuanteng Yang^1^, Yang Liu^1^, Yanxia Liu^1^, Lin Zhao^1^, Penglei Wang^1^, Yagang Zhang^1,^*, Yue Lin^2,^*, Yen Wei^3,4,^*

^1^ School of Materials and Energy, University of Electronic Science and Technology of China, Chengdu 611731, P. R. China

^2^ Hefei National Research Center for Physical Sciences at the Microscale, University of Science and Technology of China, Hefei 230026, P. R. China

^3^ The Key Laboratory of Bioorganic Phosphorus Chemistry & Chemical Biology (Ministry of Education), Department of Chemistry, Tsinghua University, Beijing 100084, P. R. China

^4^ School of Materials Science and Engineering, North Minzu University, Yinchuan 750021, P. R. China

*Corresponding authors. E-mail: [ygzhang@uestc.edu.cn](mailto:ygzhang@uestc.edu.cn) (Yagang Zhang); [linyue@ustc.edu.cn](mailto:linyue@ustc.edu.cn) (Yue Lin); [weiyen@tsinghua.edu.cn](mailto:weiyen@tsinghua.edu.cn) (Yen Wei)

**Supplementary Figures and Tables**


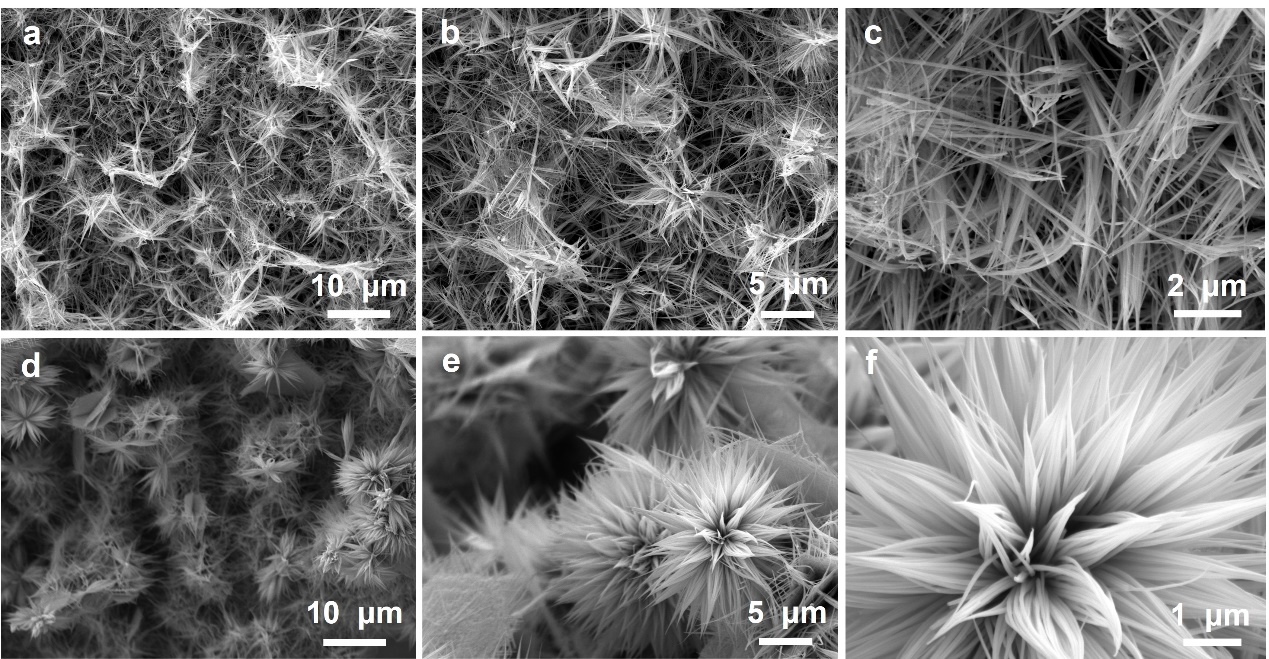


**Fig. S1** SEM images of (**a-c**) Co_3_O_4_ and (**d-f**) NiCo_2_O_4_ at different magnifications


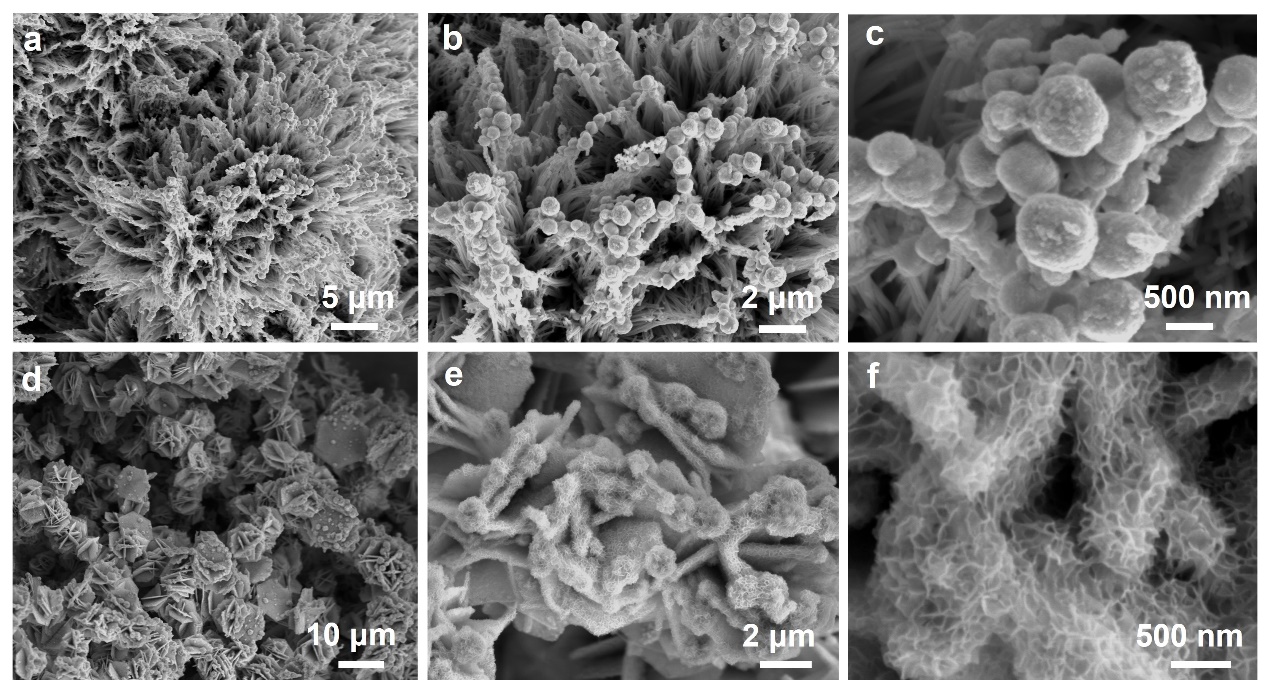


**Fig. S2** SEM images of (**a-c**) Pd-NiCo_2_O_4_-1 and (**d-f**) Pd-NiCo_2_O_4_-3 at different magnifications


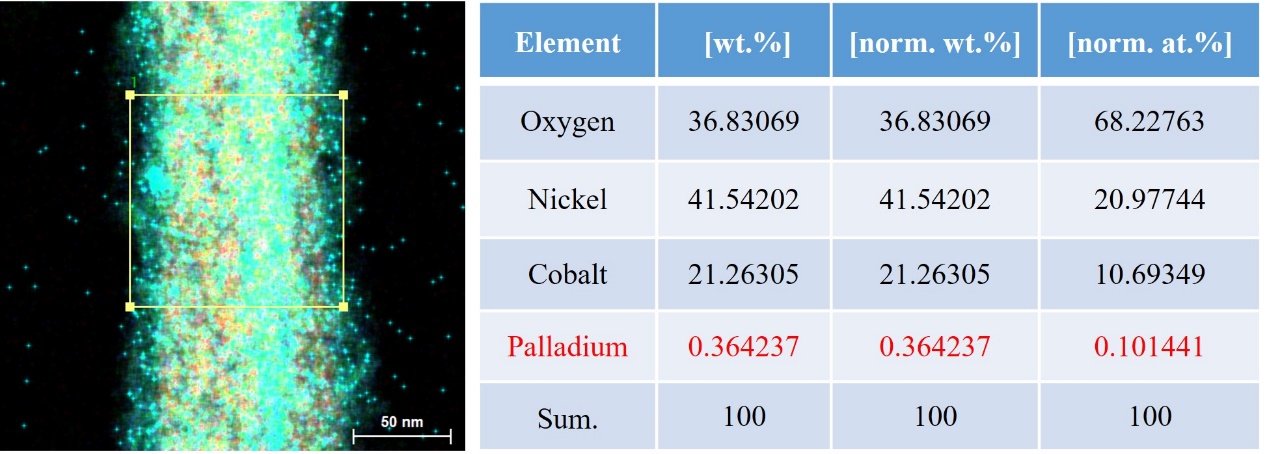


**Fig. S3** TEM-EDS of Pd-NiCo_2_O_4_

**
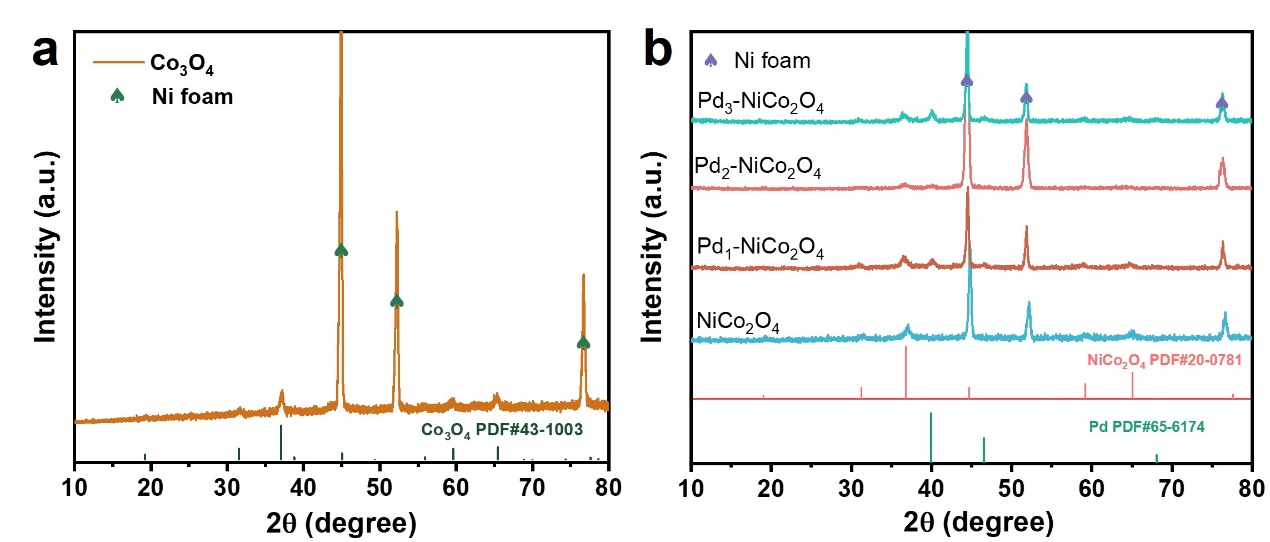
**

**Fig. S4** XRD pattern of (**a**) Co_3_O_4_ and (**b**) NiCo_2_O_4_ and Pd- NiCo_2_O_4_ with different Pd loading


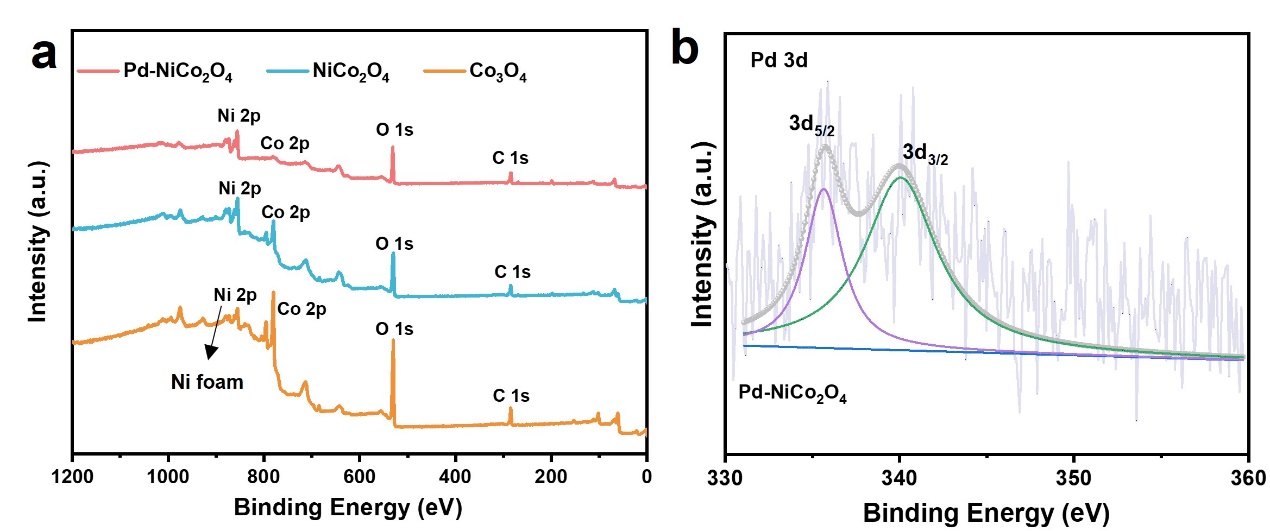


**Fig. S5** (**a**) XPS Survey spectra of Co_3_O_4_, NiCo_2_O_4_, Pd-NiCo_2_O_4_. (**b**) High-resolution XPS spectra of Pd 3d


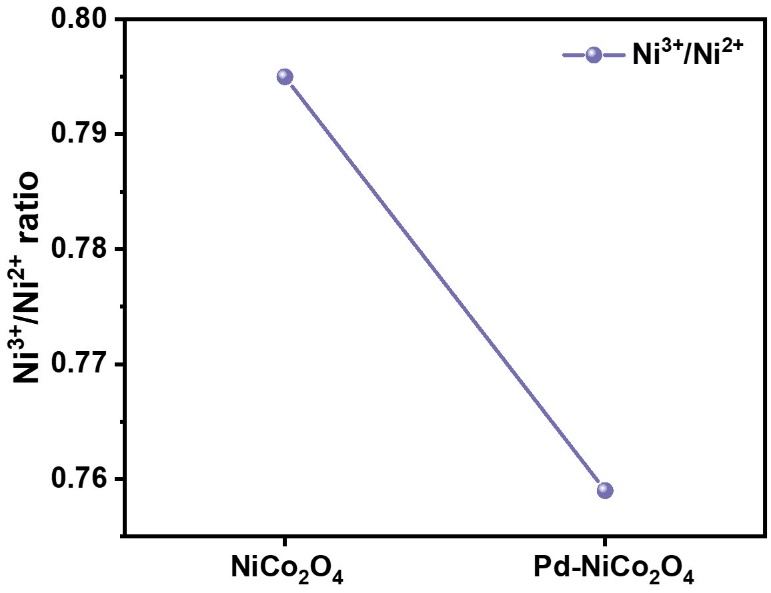


**Fig. S6** Ni^3+^/Ni^2+^ ratios obtained from XPS spectra of NiCo_2_O_4_ and Pd-NiCo_2_O_4_


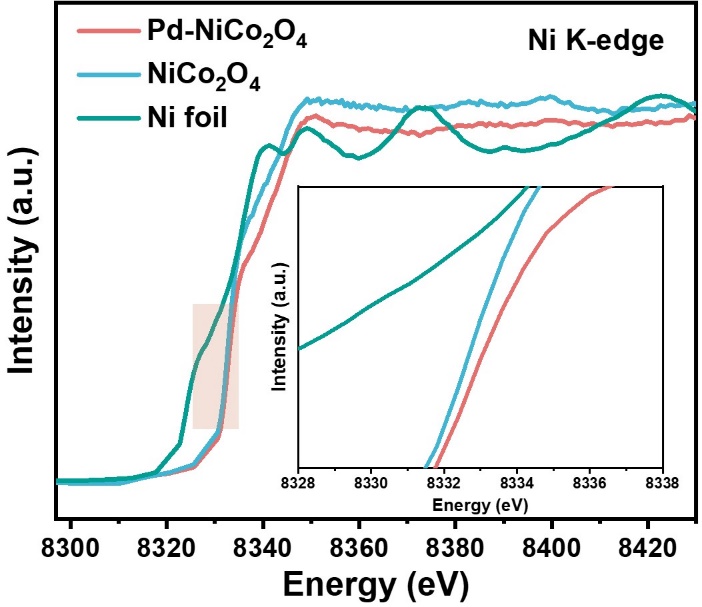


**Fig. S7** Ni K-edge XANES spectra of NiCo_2_O_4_, Pd-NiCo_2_O_4_


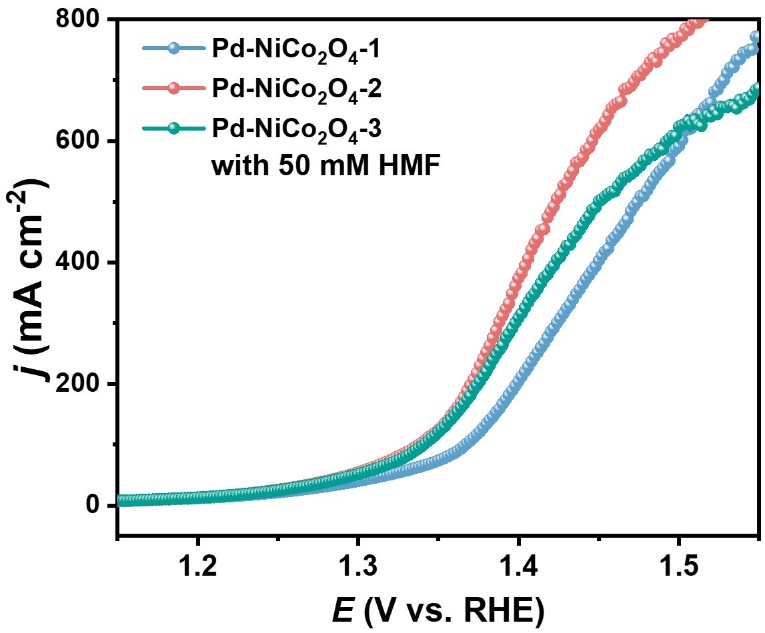


**Fig. S8** LSV curves of NiCo_2_O_4_ with various Pd loading in 1 M KOH with 50 mM HMF


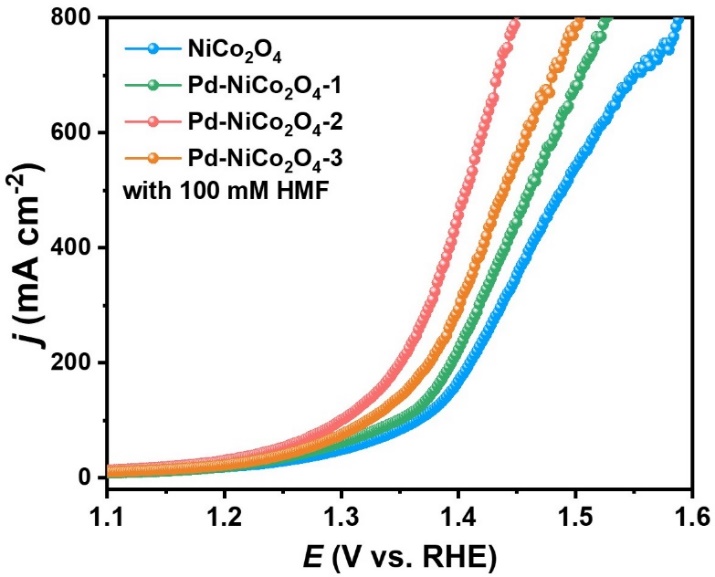


**Fig. S9** LSV curves of NiCo_2_O_4_ and NiCo_2_O_4_ with various Pd loading in 1 M KOH with 100 mM HMF


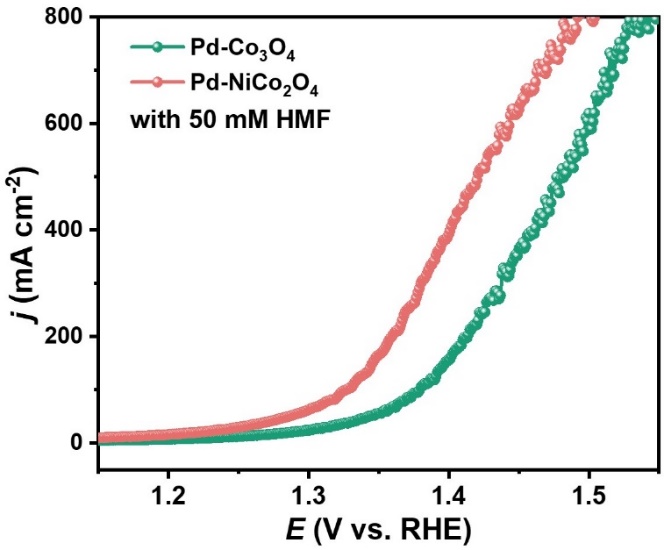


**Fig. S10** LSV curves of Pd-NiCo_2_O_4_ and Pd-Co_3_O_4_ in 1 M KOH with 50 mM HMF


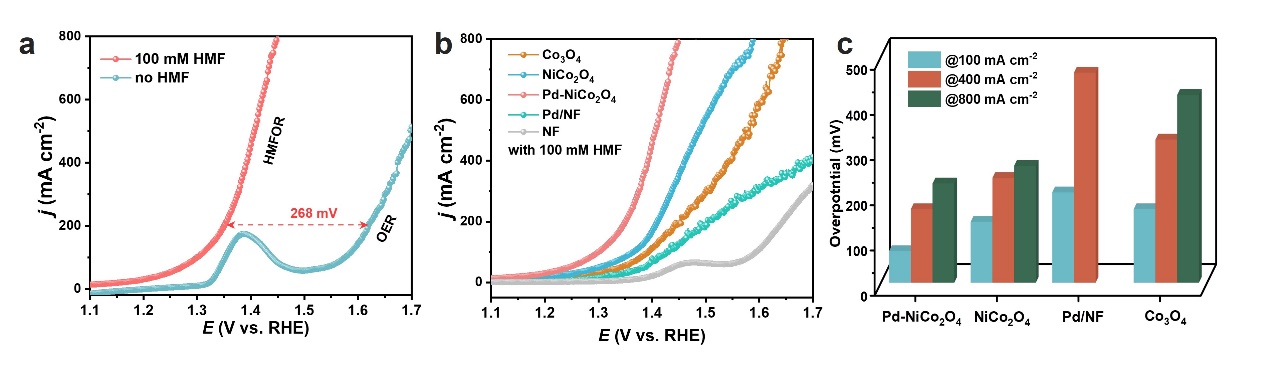


**Fig. S11 (a**) LSV curves of Pd-NiCo_2_O_4_ in 1.0 M KOH with and without 100 mM HMF. (**b**) LSV curves of different samples in 1.0 M KOH with 100 mM HMF, and (**c**) corresponding overpotential at various current densities for HMFOR


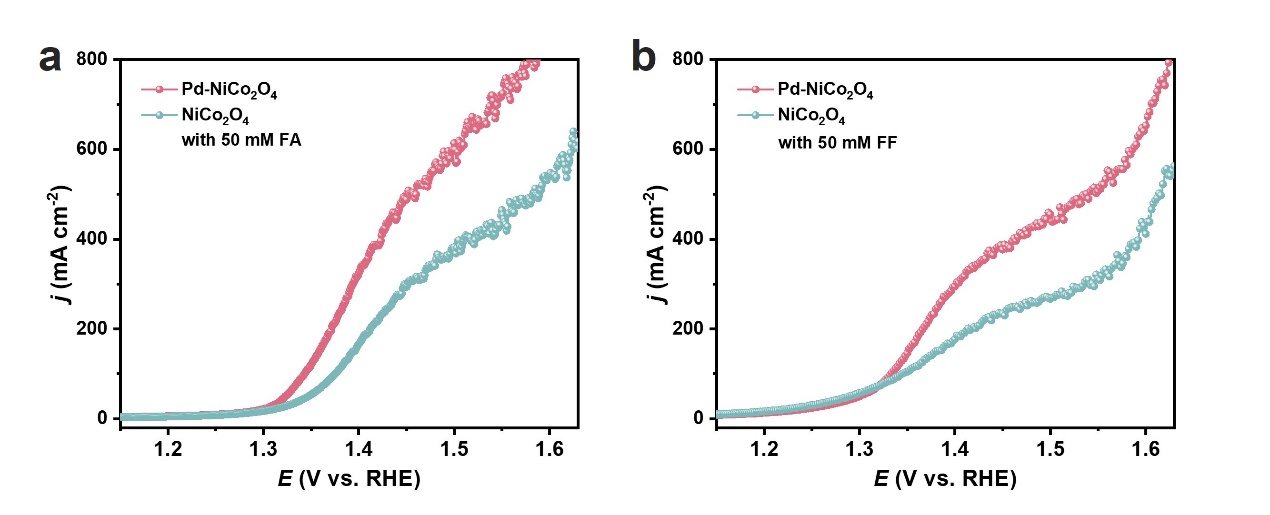


**Fig. S12** Electrochemical behavior of (a) furfuryl alcohol (R-OH) and (b) furfural (R-CHO) oxidation on Pd-NiCo_2_O_4_ and NiCo_2_O_4_ electrodes.


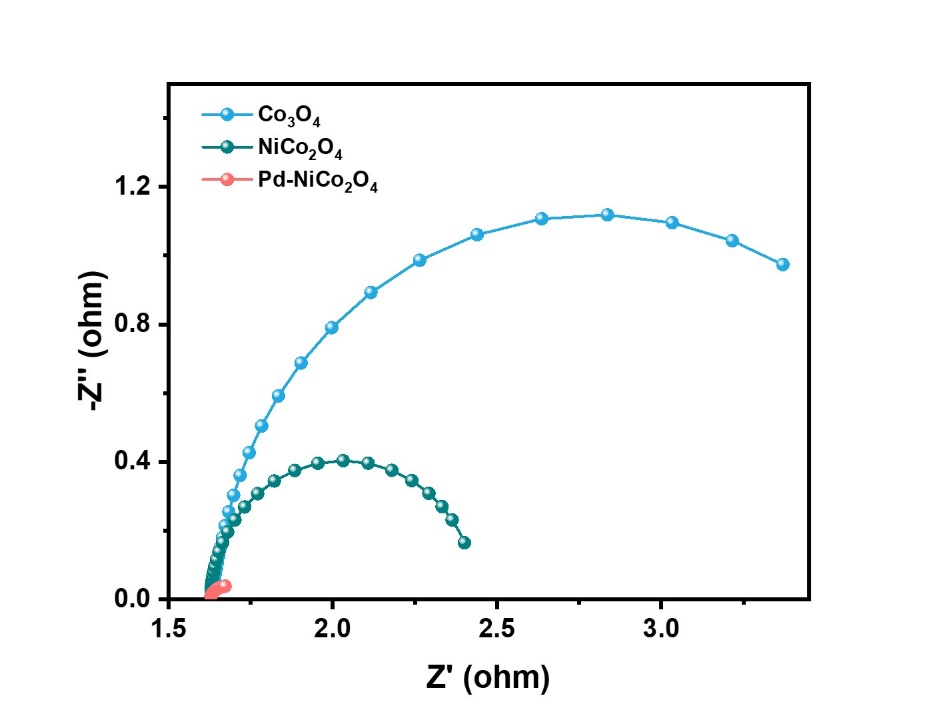


**Fig. S13** Nyquist plots of Co_3_O_4_, NiCo_2_O_4_, and Pd-NiCo_2_O_4_


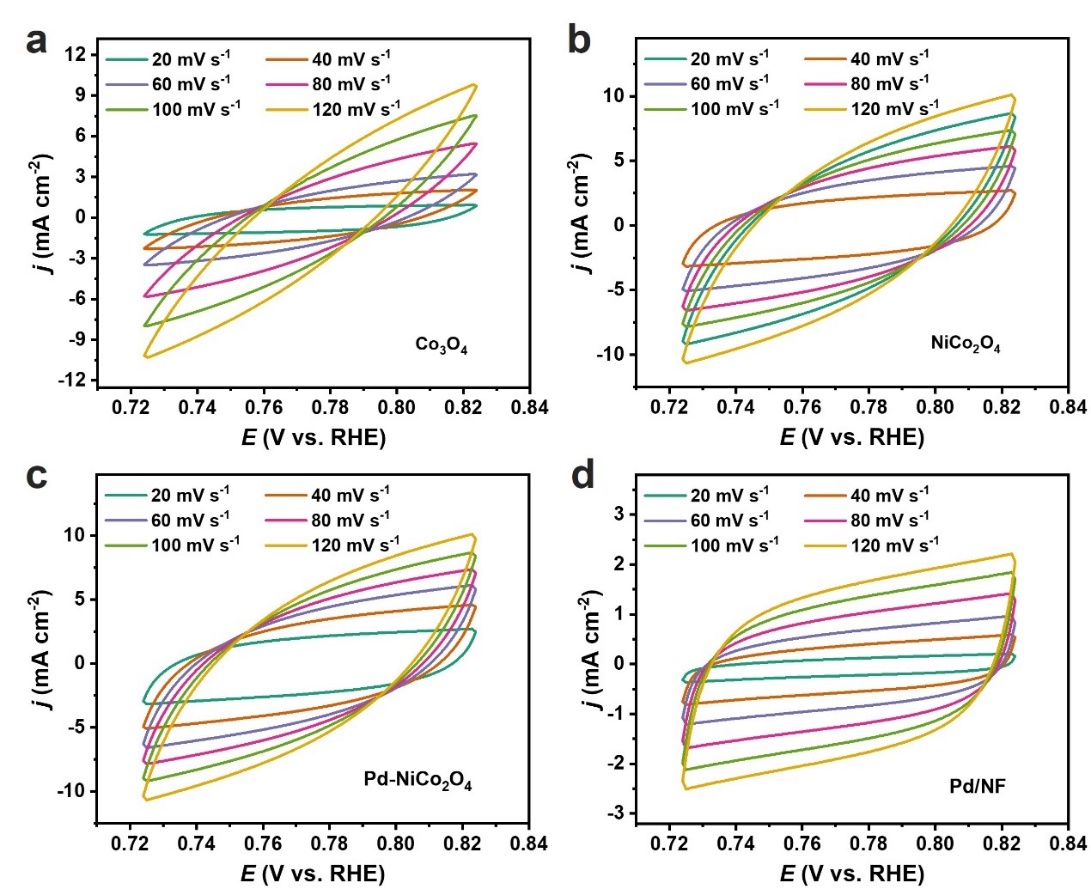


**Fig. S14** Cyclic voltammograms (CVs) curves of (**a**) Co_3_O_4_, (**b**) NiCo_2_O_4_, (**c**) Pd-NiCo_2_O_4_ and (**d**) Pd/NF at different scan rates increasing from 20 to 120 mV s^–1^ with an interval point of 20 mV s^–1^

**
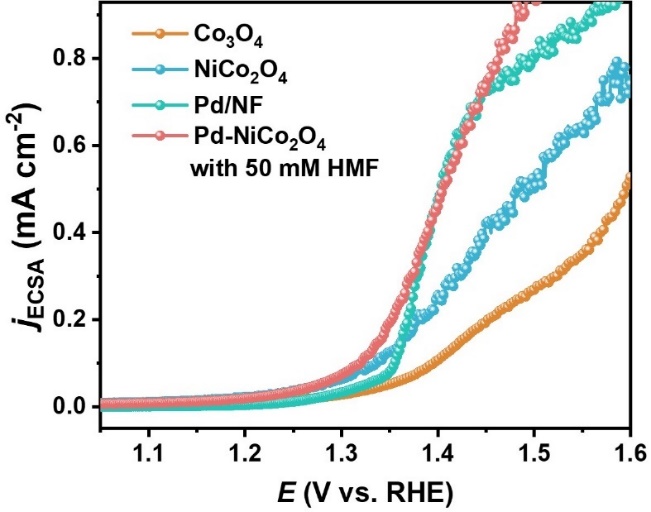
**

**Fig. S15** ECSA-normalized LSV curves of samples in 1 M KOH with 50 mM HMF


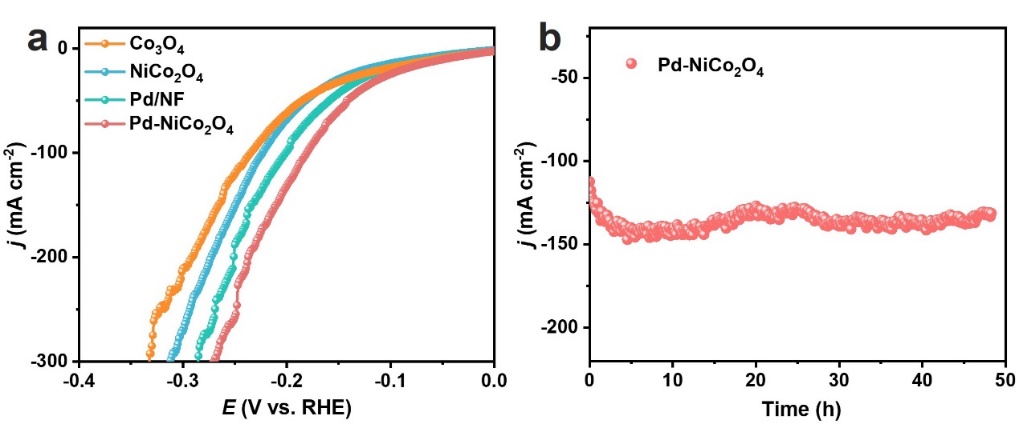


**Fig. S16** (**a**) HER LSV curves of samples and (**b**) stability (I-t) test at –150 mA cm^–2^


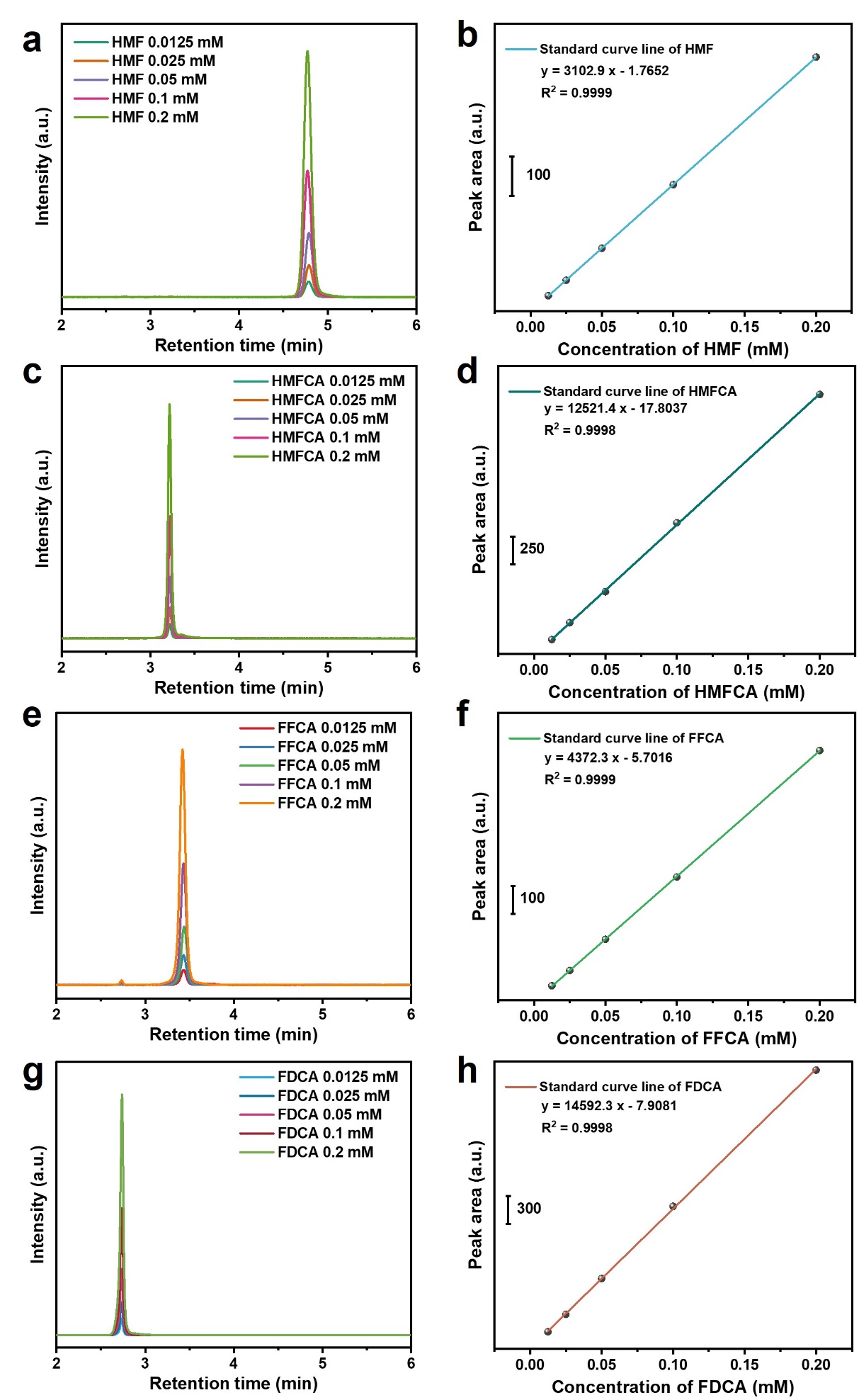


**Fig. S17** HPLC standard curve measurements of pure (**a, b**) HMF, (**c, d**) HMFCA, (**e, f**) FFCA, (**g, h**) FDCA


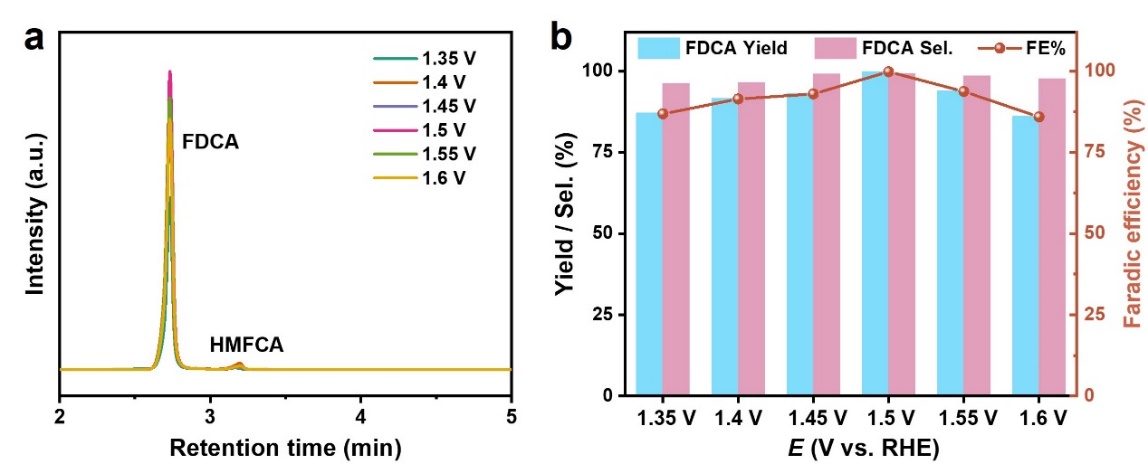


**Fig. S18** (**a**) HPLC curve measurements (**b**) the FDCA selectivity and Faradaic efficiency at different potentials for Pd-NiCo_2_O_4_


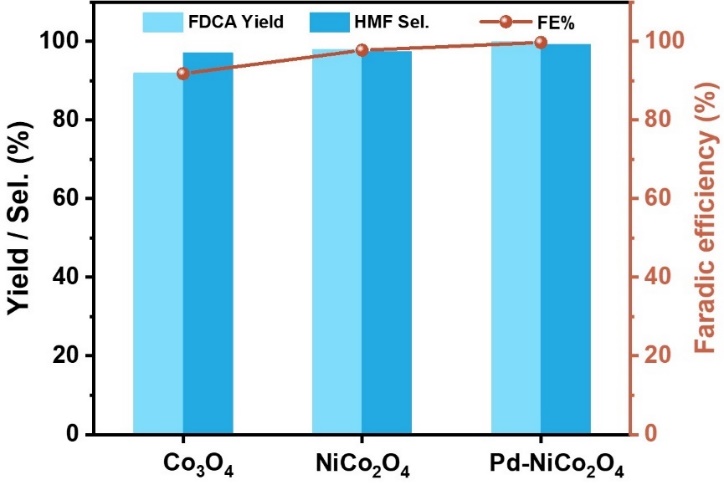


**Fig. S19** HMF conversion, FDCA yield and Faradaic efficiency for different samples


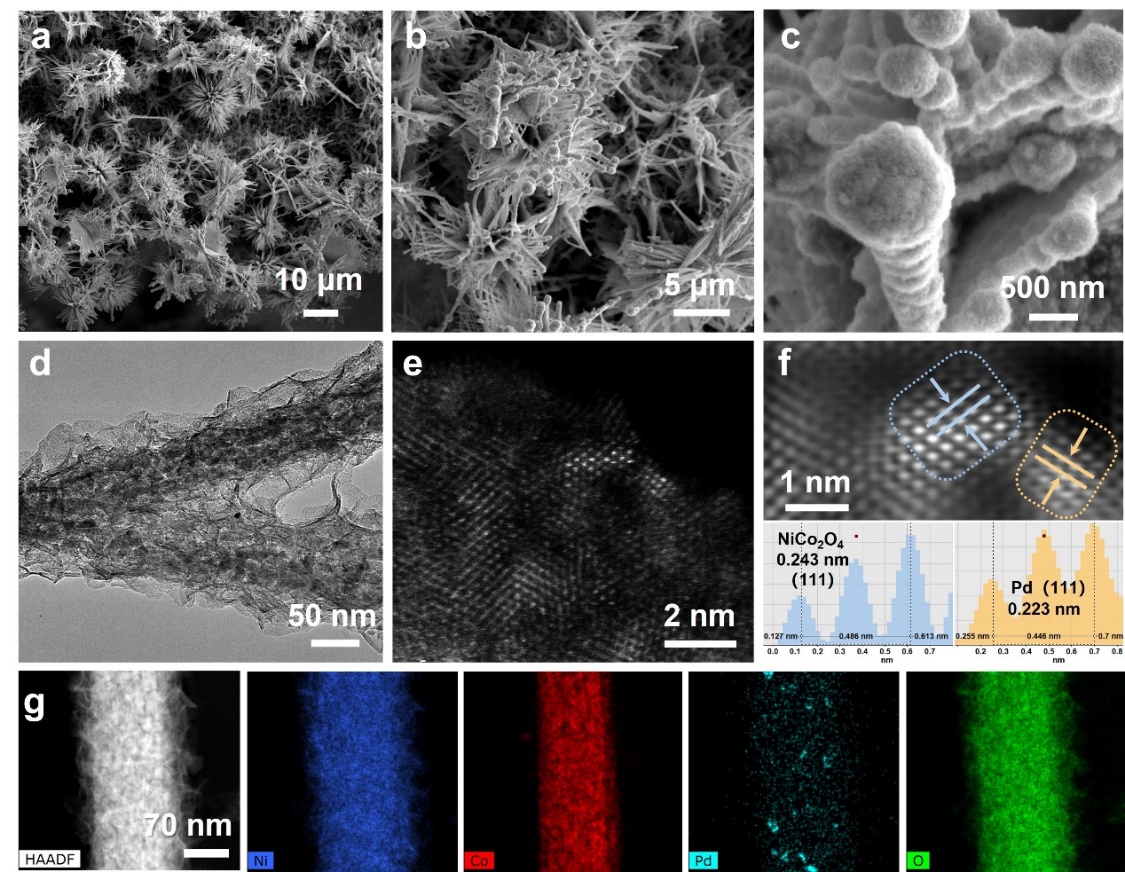


**Fig. S20 (a-c**) SEM images (**d**) TEM, (**e, f**) HAADF-STEM images and (**g**) STEM-mapping of Pd-NiCo_2_O_4_ after HMFOR


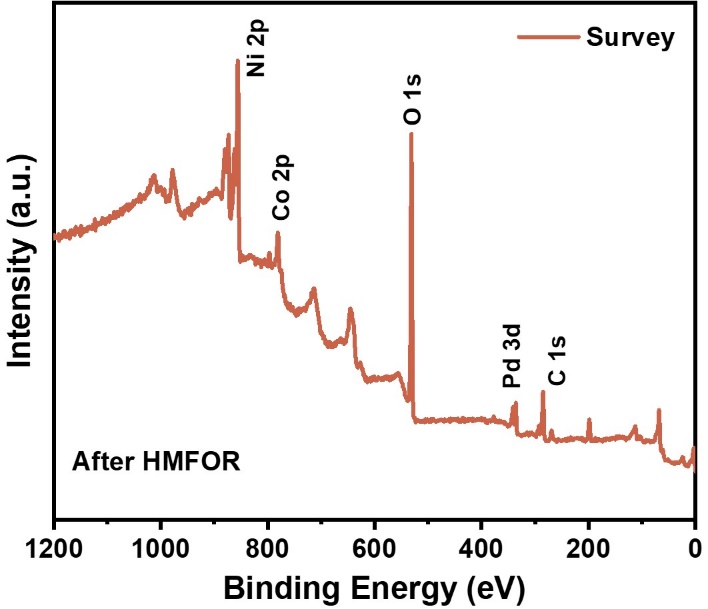


**Fig. S21** XPS Survey spectrum of Pd-NiCo_2_O_4_ after HMFOR


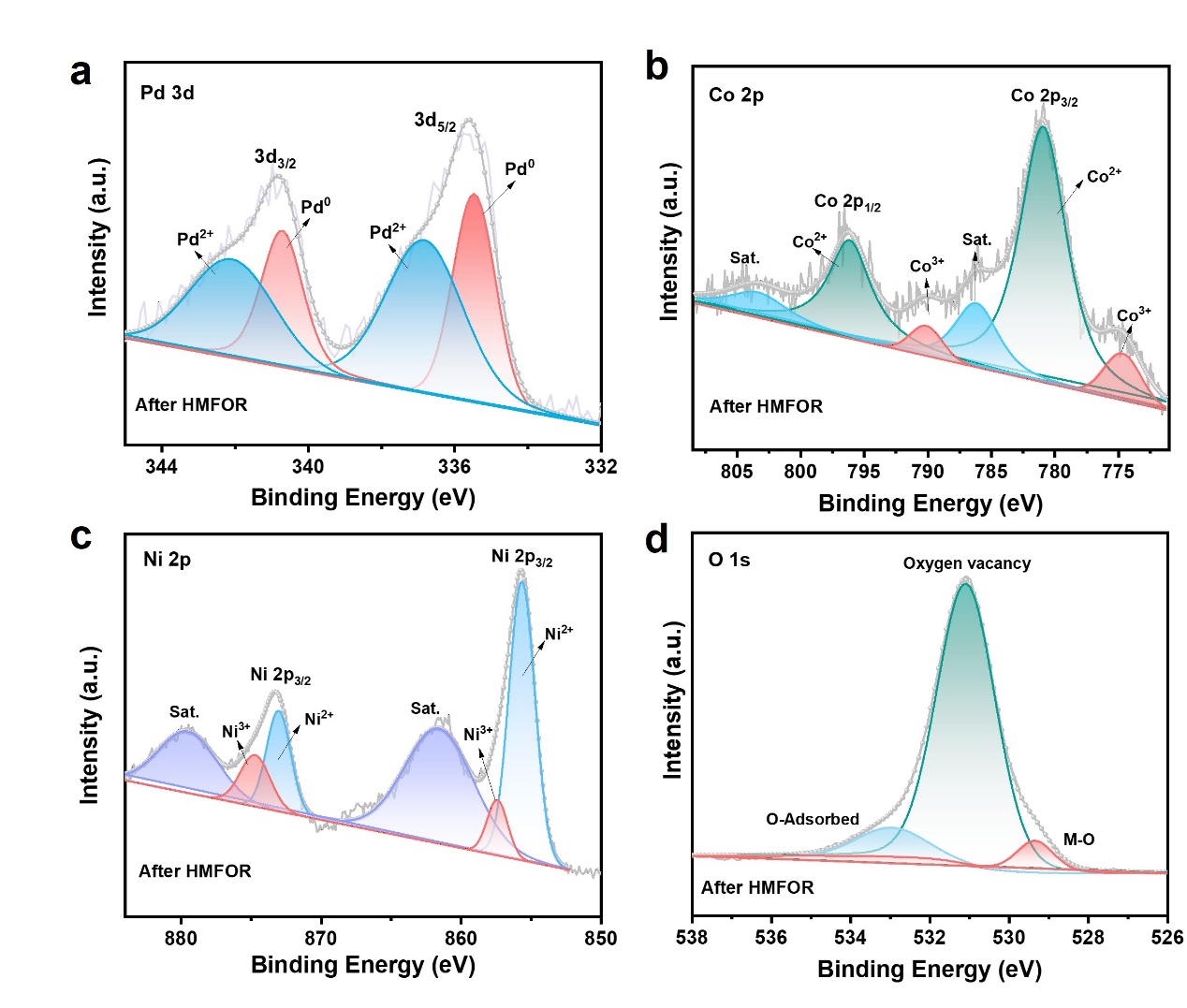


**Fig. S22** High-resolution (**a**) Pd 3d, (**b**) Co 2p, (**c**) Ni 2p, (**d**) O 1s XPS spectra of Pd-NiCo_2_O_4_ after HMFOR


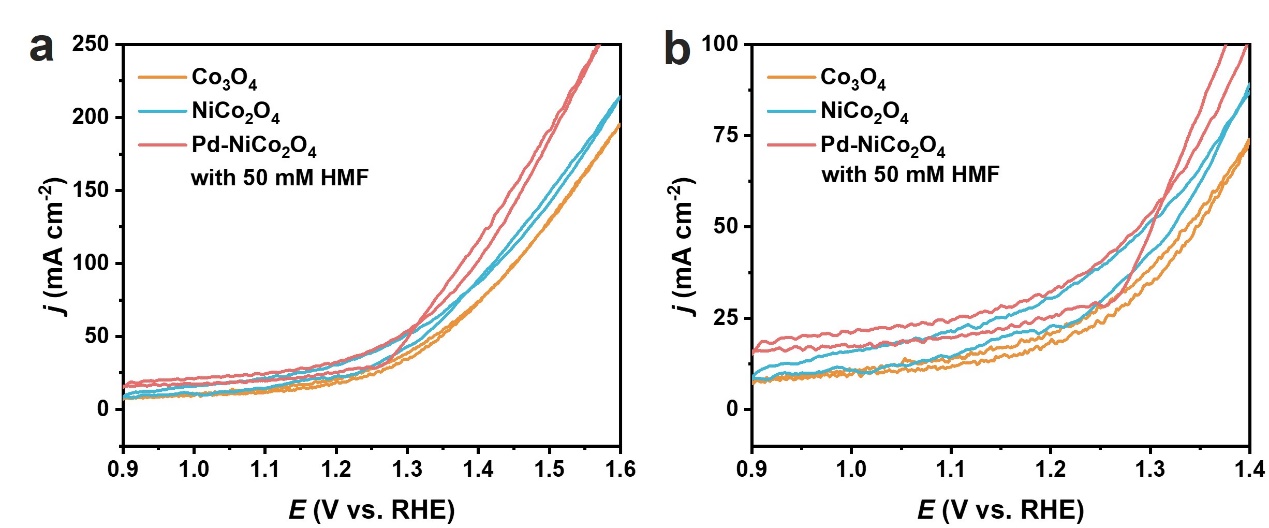


**Fig. S23** CV curves of Co_3_O_4_, NiCo_2_O_4_, and Pd-NiCo_2_O_4_ in 1 M KOH with 50 mM HMF


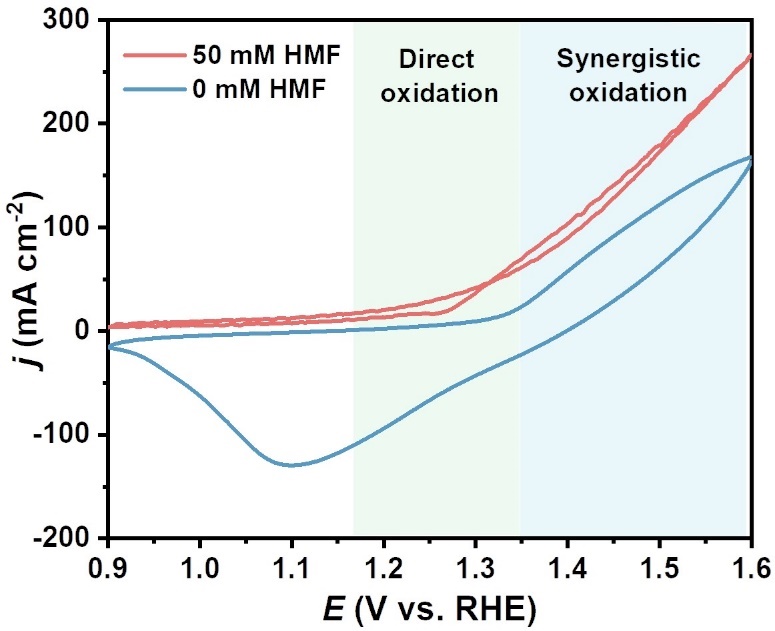


**Fig. S24** CV curves of Pd-NiCo_2_O_4_ in 1 M KOH with and without 50 mM HMF


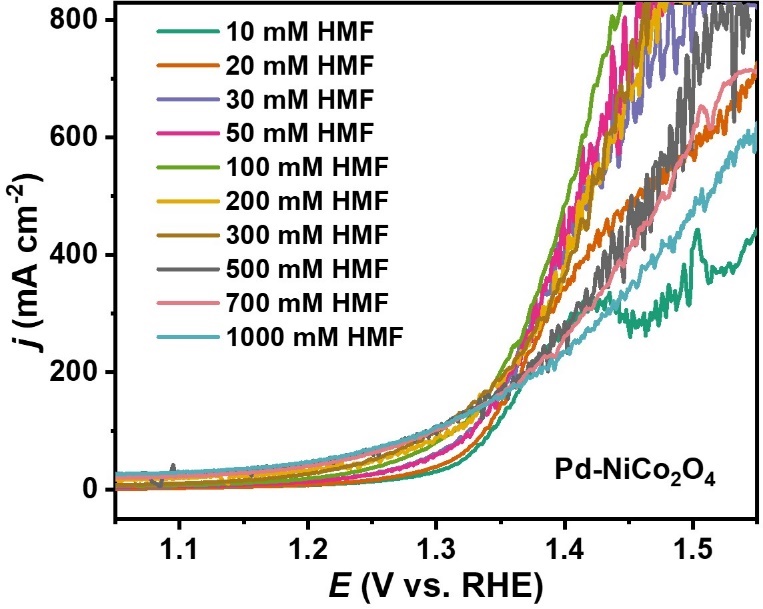


**Fig. S25** LSV curves of Pd-NiCo_2_O_4_ in 1 M KOH with different HMF concentration

**
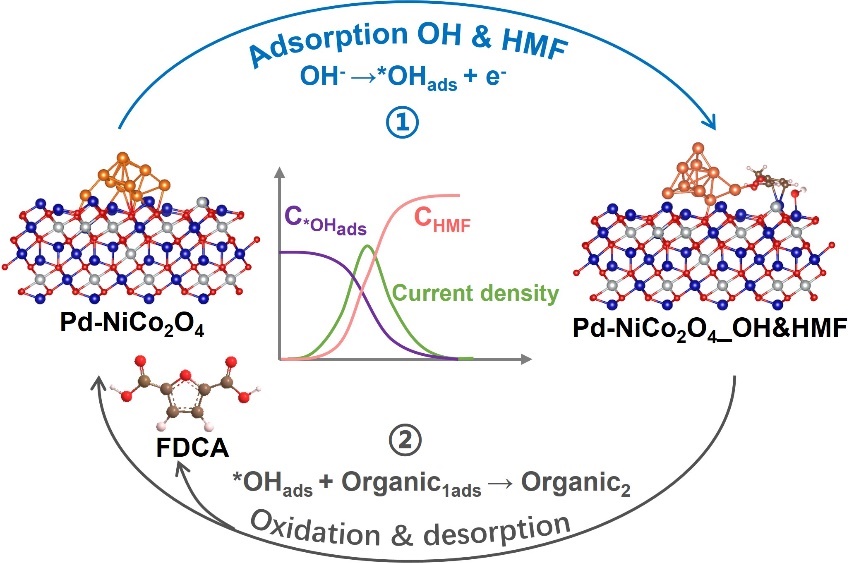
**

**Fig. S26** Schematic representation of the direct oxidation of HMF

**
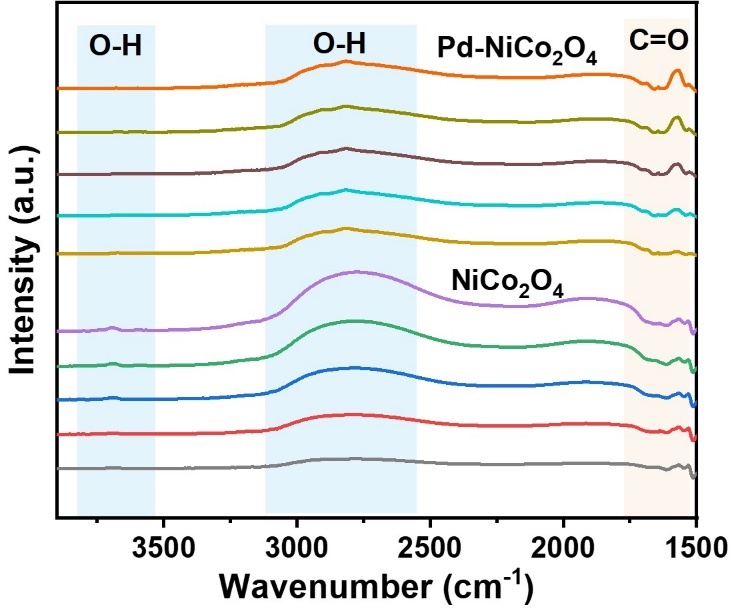
**

**Fig. S27** In situ ATR-FTIR spectra over Pd-NiCo_2_O_4_ and NiCo_2_O_4_ samples


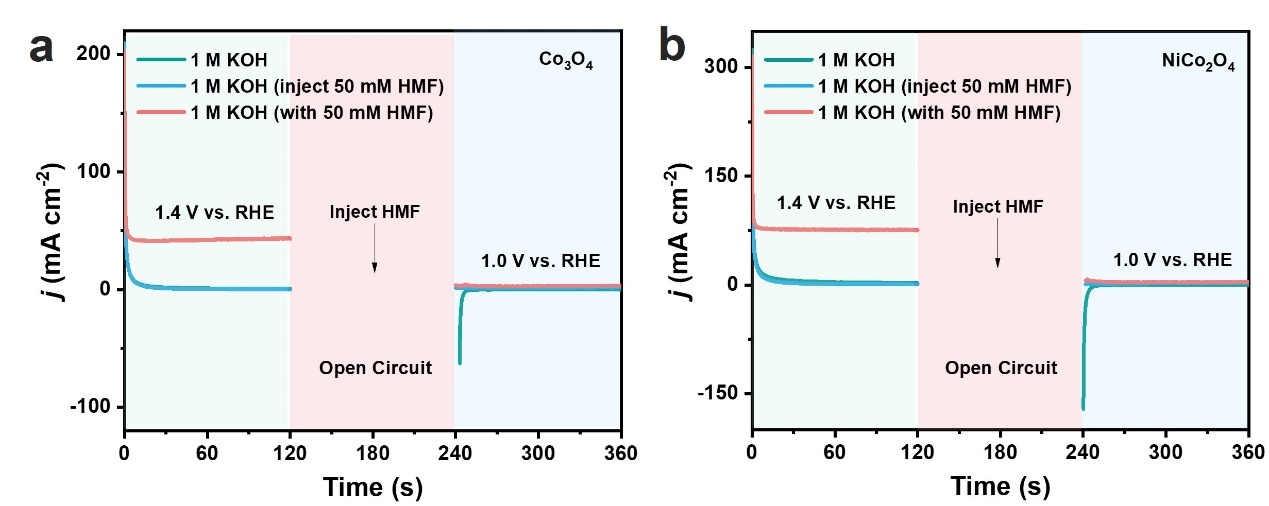


**Fig. S28** Multipotential-step curves of and (**a**) Co_2_O_4_ and (**b**) NiCo_2_O_4_ in 1 M KOH solution with and without 50 mM HMF addition


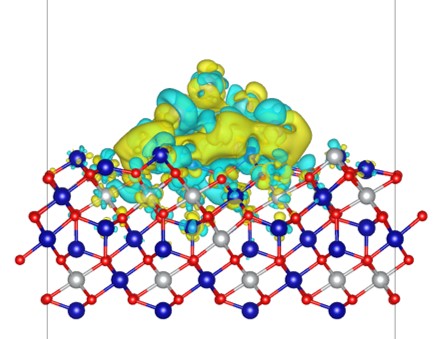


**Fig. S29** Charge density difference for Pd-NiCo_2_O_4_


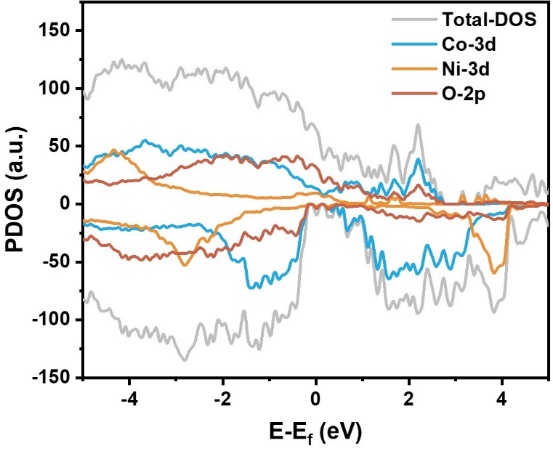


**Fig. S30** PDOS of NiCo_2_O_4_


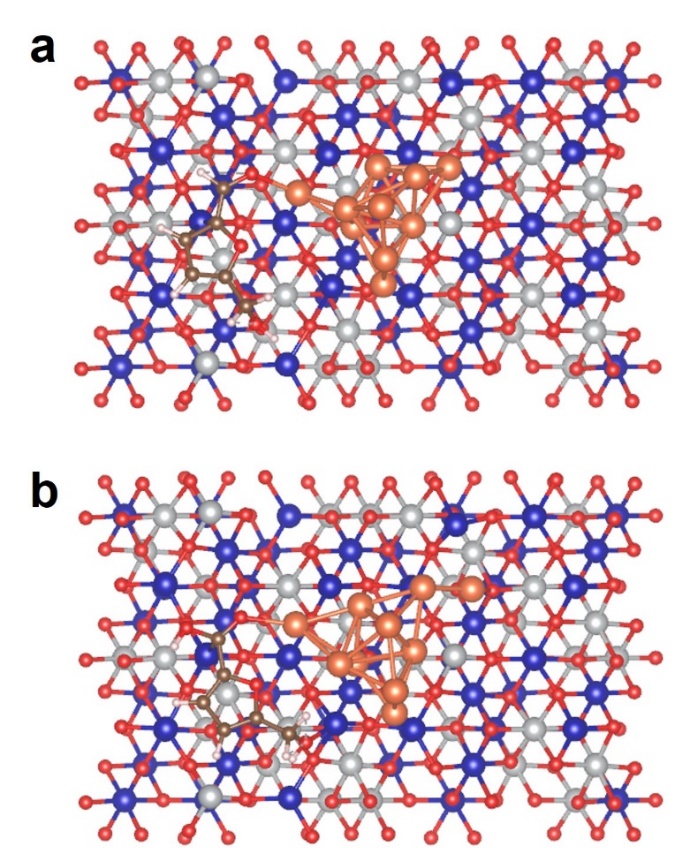


**Fig. S31** Optimized stable adsorption configures (top review) for HMF and HMFCA on Pd-NiCo_2_O_4_


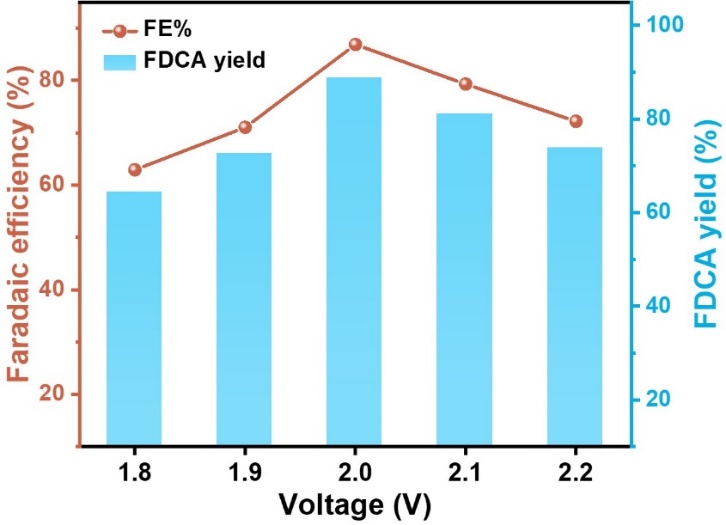


**Fig. S32** FDCA yield and Faradaic efficiency of Pd-NiCo_2_O_4_ in coupled two-electrode cell at different voltages


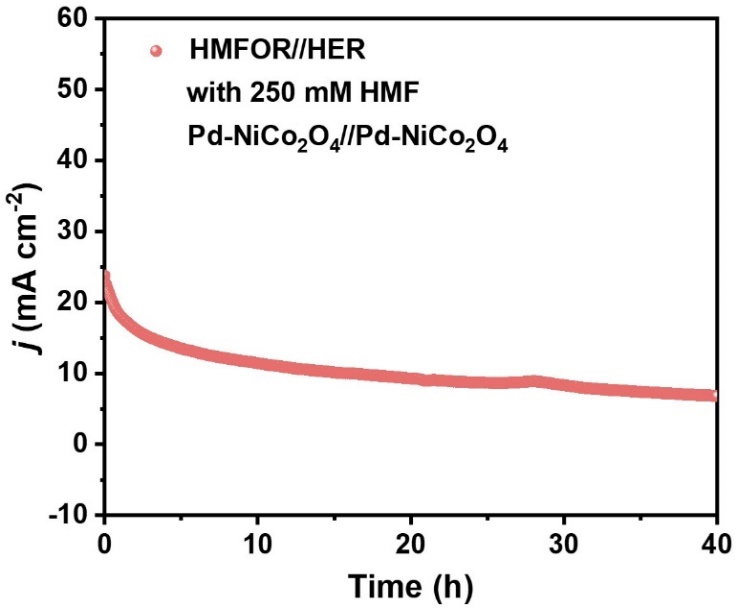


**Fig. S33** Long-term test of Pd-NiCo_2_O_4_ in two-electrode cell

**Table S1** ICP results of Co, Ni and Pd elements in Pd-NiCo_2_O_4_

| **Element** | **Wt%** | **At%** |
| --- | --- | --- |
| Co | 19.54 | 0.33 |
| Ni | 32.94 | 0.56 |
| Pd | 10.80 | 0.10 |

**Table S2** XPS results of Pd-NiCo_2_O_4_

| **Name** | **Atomic%** |  | **PP Hgt (N)** | **PP At%** |
| --- | --- | --- | --- | --- |
| C 1s | 35.23 |  | 31.88 | 40.52 |
| Co 2p | 2.9 |  | 0.56 | 0.71 |
| Ni 2p | 18.67 |  | 5.44 | 6.91 |
| O 1s | 42.7 |  | 40.65 | 51.67 |
| Pd 3d | 0.5 |  | 0.15 | 0.19 |

**Table S3** XPS fitting results of samples

| Catalyst | Binding energy (eV) | | | | | | | |
| --- | --- | --- | --- | --- | --- | --- | --- | --- |
|  | **Co^2+^** | | **Co^3+^** | | **Ni^2+^** | | **Ni^3+^** | |
| Co_3_O_4_ | 796.38 | 781.46 | 794.80 | 779.83 | / | / | / | / |
| NiCo_2_O_4_ | 796.45 | 781.64 | 794.67 | 779.76 | 872.54 | 854.62 | 874.59 | 856.41 |
| Pd-NiCo_2_O_4_ | 797.28 | 781.29 | 792.73 | 775.42 | 873.32 | 855.64 | 874.92 | 857.09 |

**Table S4** Comparison of the HMFOR performance between Pd-NiCo_2_O_4_ and recently reported advanced catalysts

| Catalysts | C_HMF_  (mM) | Potential  (V vs. RHE) | Current density  (mA cm^-2^) | Faradaic efficiency (FE, %) | References |
| --- | --- | --- | --- | --- | --- |
| NiFe-1 | 50 | 1.5 | 630 | 92 | [S1] |
| CoOOH/CF | 50 | 1.5 | 420 | 99 | [S2] |
| CuCo_2_O_4_ | 50 | 1.5 | 225 | 94 | [S3] |
| Pd/NiCo | 50 | 1.5 | 81 | 95.9 | [S4] |
| Pt/Ni(OH)_2_ | 50 | 1.6 | 38 | 98.7 | [S5] |
| NiFeLDH/CPF | 10 | 1.45 | 20 | 99.4 | [S6] |
| Co_0.4_NiS@NF | 50 | 1.45 | 497 | 99.1 | [S7] |
| NF@Co_3_O_4_/CeO_2_ | 50 | 1.4 | 32 | 97.5 | [S8] |
| NiS_x_/β-Ni(OH)_2_/Ni | 50 | 1.38  1.4 | 120  392 | 98.3 | [S9] |
| Pd-NiCo_2_O_4_ | 50 | 1.44  1.5 | 596  800 | 99.6 | **This work** |

**Table S5** Comparison of catalytic performance of the state-of-the-art catalysts reported in literatures and the catalyst in this work

| Catalysts | HMFOR//HER | | | HMFOR performance | | References |
| --- | --- | --- | --- | --- | --- | --- |
|  | E*_j_*_10_ (V) | E*_j_*_50_ (V) | *j*_F.E._ | E*_j_*_100_ (V) | F.E. (%) |  |
| MoO_2_-FeP@C | 1.49 | 1.6 | 20 | 1.42 | 97.8 | [S10] |
| E-CoAl-LDH-NSA | 1.5 | 1.74 | - | 1.59 | 99.4 | [S11] |
| NF@Mo-Ni_0.85_Se | 1.4 | 1.5 | - | 1.46 | 95 | [S12] |
| Cu_x_S@NiCo-LDH | 1.34 | 1.49 | - | 1.36 | 99 | [S13] |
| NiSe/NiO_x_ | 1.56 | 1.65 | - | 1.35 | 99 | [S14] |
| NiCu NTs | 1.3 | 1.47 | - | 1.38 | 96.4 | [S15] |
| NiCo_2_@MoO_2_/NF | 1.25 | 1.48 | - | 1.27 | 99.2 | [S16] |
| Rh-O_5_-Ni(Fe) | 1.32 | 1.42 | 40 | 1.39 | 98.5 | [S17] |
| Co_9_S_8_@Ni_3_S_2_/NF | 1.42 | 1.61 | - | 1.42 | 98.4 | [S18] |
| Pd-NiCo_2_O_4_ | 1.07 | 1.40 | 100 | 1.32 | 99.6 | **This work** |

**Supplementary References**

1. C. Wang, Y. Wu, A. Bodach, M. L. Krebs, W. Schuhmann et al. A novel electrode for value-generating anode reactions in water electrolyzers at industrial current densities. Angew. Chem., Int. Ed. **62**(7), e202215804 (2023). <https://doi.org/https://doi.org/10.1002/anie.202215804>
2. R. Zhang, S. Jiang, Y. Rao, S. Chen, Q. Yue et al. Electrochemical biomass upgrading on CoOOH nanosheets in a hybrid water electrolyzer. Green Chem. **23**(6), 2525-2530 (2021). <https://doi.org/10.1039/D0GC04157B>
3. Y. Lu, C.-L. Dong, Y.-C. Huang, Y. Zou, Z. Liu et al. Identifying the geometric site dependence of spinel oxides for the electrooxidation of 5-hydroxymethylfurfural. Angew. Chem., Int. Ed. **59**(43), 19215-19221 (2020). <https://doi.org/https://doi.org/10.1002/anie.202007767>
4. G. Liu, T. Nie, Z. Song, X. Sun, T. Shen et al. Pd loaded nico hydroxides for biomass electrooxidation: Understanding the synergistic effect of proton deintercalation and adsorption kinetics. Angew. Chem., Int. Ed. **62**(45), e202311696 (2023). <https://doi.org/https://doi.org/10.1002/anie.202311696>
5. B. Zhou, Y. Li, Y. Zou, W. Chen, W. Zhou et al. Platinum modulates redox properties and 5-hydroxymethylfurfural adsorption kinetics of Ni(OH)_2_ for biomass upgrading. Angew. Chem., Int. Ed. **60**(42), 22908-22914 (2021). <https://doi.org/https://doi.org/10.1002/anie.202109211>
6. W.-J. Liu, L. Dang, Z. Xu, H.-Q. Yu, S. Jin et al. Electrochemical oxidation of 5-hydroxymethylfurfural with NiFe layered double hydroxide (LDH) nanosheet catalysts. ACS Cataly. **8**(6), 5533-5541 (2018). <https://doi.org/10.1021/acscatal.8b01017>
7. Y. Sun, J. Wang, Y. Qi, W. Li, C. Wang. Efficient electrooxidation of 5-hydroxymethylfurfural using Co-doped Ni_3_S_2_ catalyst: Promising for H_2_ production under industrial-level current density. Adv. Sci. **9**(17), 2200957 (2022). <https://doi.org/https://doi.org/10.1002/advs.202200957>
8. G. Zhao, G. Hai, P. Zhou, Z. Liu, Y. Zhang et al. Electrochemical oxidation of 5-hydroxymethylfurfural on CeO_2_-modified Co_3_O_4_ with regulated intermediate adsorption and promoted charge transfer. Adv. Funct. Mater. **33**(14), 2213170 (2023). <https://doi.org/https://doi.org/10.1002/adfm.202213170>
9. C. Liu, X.-R. Shi, K. Yue, P. Wang, K. Zhan et al. S-species-evoked high-valence Ni^2+δ^ of the evolved β-Ni(OH)_2_ electrode for selective oxidation of 5-hydroxymethylfurfural. Adv. Mater. **35**(12), 2211177 (2023). <https://doi.org/https://doi.org/10.1002/adma.202211177>
10. G. Yang, Y. Jiao, H. Yan, Y. Xie, A. Wu et al. Interfacial engineering of MoO_2_-FeP heterojunction for highly efficient hydrogen evolution coupled with biomass electrooxidation. Adv. Mater. **32**(17), 2000455 (2020). <https://doi.org/https://doi.org/10.1002/adma.202000455>
11. Y. Song, Z. Li, K. Fan, Z. Ren, W. Xie et al. Ultrathin layered double hydroxides nanosheets array towards efficient electrooxidation of 5-hydroxymethylfurfural coupled with hydrogen generation. Appl. Catal., B. **299**(120669 (2021). <https://doi.org/https://doi.org/10.1016/j.apcatb.2021.120669>
12. C. Yang, C. Wang, L. Zhou, W. Duan, Y. Song et al. Refining d-band center in Ni_0.85_Se by Mo doping: A strategy for boosting hydrogen generation via coupling electrocatalytic oxidation 5-hydroxymethylfurfural. Chem. Eng. J. **422**(130125 (2021). <https://doi.org/https://doi.org/10.1016/j.cej.2021.130125>
13. X. Deng, X. Kang, M. Li, K. Xiang, C. Wang et al. Coupling efficient biomass upgrading with H_2_ production via bifunctional Cu_x_S@NiCo-LDH core–shell nanoarray electrocatalysts. J. Mater. Chem. A **8**(3), 1138-1146 (2020). <https://doi.org/10.1039/C9TA06917H>
14. L. Gao, Z. Liu, J. Ma, L. Zhong, Z. Song et al. NiSe@NiO_x_ core-shell nanowires as a non-precious electrocatalyst for upgrading 5-hydroxymethylfurfural into 2,5-furandicarboxylic acid. Appl. Catal., B. **261**, 118235 (2020). <https://doi.org/https://doi.org/10.1016/j.apcatb.2019.118235>
15. L. Zheng, Y. Zhao, P. Xu, Z. Lv, X. Shi et al. Biomass upgrading coupled with H_2_ production via a nonprecious and versatile cu-doped nickel nanotube electrocatalyst. J. Mater. Chem. A **10**(18), 10181-10191 (2022). <https://doi.org/10.1039/D2TA00579D>
16. J. Wu, J. Chen, T. Yu, Z. Zhai, Y. Zhu et al. Boosting electrochemical kinetics of NiCo_2_ via MoO_2_ modification for biomass upgrading assisted hydrogen evolution. ACS Catal. **13**(20), 13257-13266 (2023). <https://doi.org/10.1021/acscatal.3c03094>
17. L. Zeng, Y. Chen, M. Sun, Q. Huang, K. Sun et al. Cooperative Rh-O_5_/Ni(Fe) site for efficient biomass upgrading coupled with H_2_ production. J. Am. Chem. Soc. **145**(32), 17577-17587 (2023). <https://doi.org/10.1021/jacs.3c02570>
18. R. Zhang, F. Gao, C. Yang, Y. Bian, G. Wang et al. Boosting hydrogen evolution via anodic oxidation of 5-hydroxymethylfurfural in anion exchange membrane electrolyzer over a metallic heterostructure. Mater. Today Nano **23**, 100373 (2023). <https://doi.org/https://doi.org/10.1016/j.mtnano.2023.100373>
